# Mechano-immune interactions in musculoskeletal aging: Mechanisms and translational perspectives

**DOI:** 10.7150/thno.131071

**Published:** 2026-06-17

**Authors:** Fulin Zhou, Xianglong Zhou, Jiheng Xiao, Jianhui Xiang, Yang Liu, Weicheng Chen, Hanhong Fang, Haoran Zhou, Xiao Lv, Liming Xiong

**Affiliations:** Department of Orthopedics, Union Hospital, Tongji Medical College, Huazhong University of Science and Technology, Wuhan, Hubei Province 430022, China.

**Keywords:** aging, mechano-immunology, mechanobiology, musculoskeletal system, degenerative diseases, mechanotherapy

## Abstract

Mechano-immunology is an interdisciplinary field that examines how mechanical forces—including endogenous tissue stresses and exogenous stimuli—modulate immune cell function in the maintenance of homeostasis and the progression of disease. As the primary load-bearing framework of the body, the musculoskeletal system depends on coordinated mechanical loading to sustain immune balance and tissue repair. With aging, reduced physical activity, progressive matrix stiffening, and impaired mechanosensing in immune-cells converge to disrupt this mechano-immune axis, undermining immune clearance, inflammatory regulation, and regenerative capacity within musculoskeletal tissues. These alterations collectively contribute to the onset and progression of osteoarthritis, osteoporosis, and other degenerative musculoskeletal disorders. This review summarizes current advances in understanding the mechanisms of mechano-immunology within the musculoskeletal system under physiological conditions and delineates its involvement in the initiation and progression of degenerative diseases. Emerging strategies, including exercise-based interventions, controlled mechanical stimulation, and biomaterial engineering approaches targeting mechano-immune pathways, are also discussed as potential means to restore musculoskeletal homeostasis during aging.

## Introduction

With the acceleration of global population aging, the incidence of degenerative musculoskeletal diseases—including osteoporosis (OP), osteoarthritis (OA), intervertebral disc degeneration (IDD), and sarcopenia—has risen significantly. These conditions substantially impair quality of life among older adults and impose an increasing burden on healthcare systems and society 1-3. Mechanical imbalance and chronic inflammation have traditionally been considered as primary contributors to disease progression. However, neither factor alone adequately explained the complex and multifaceted nature of age-related musculoskeletal degeneration 4-7. The emerging discipline of mechano-immunology provides a unifying conceptual framework to address this gap. By elucidating the bidirectional interplay between mechanical inputs and immune cell behavior, mechano-immunology offers insight into how aging-associated alterations in tissue stiffness, mechanosensing capacity of immune cells, and mechanical load distribution reshape immune regulation and drive musculoskeletal degeneration 8.

**Figure 1:**
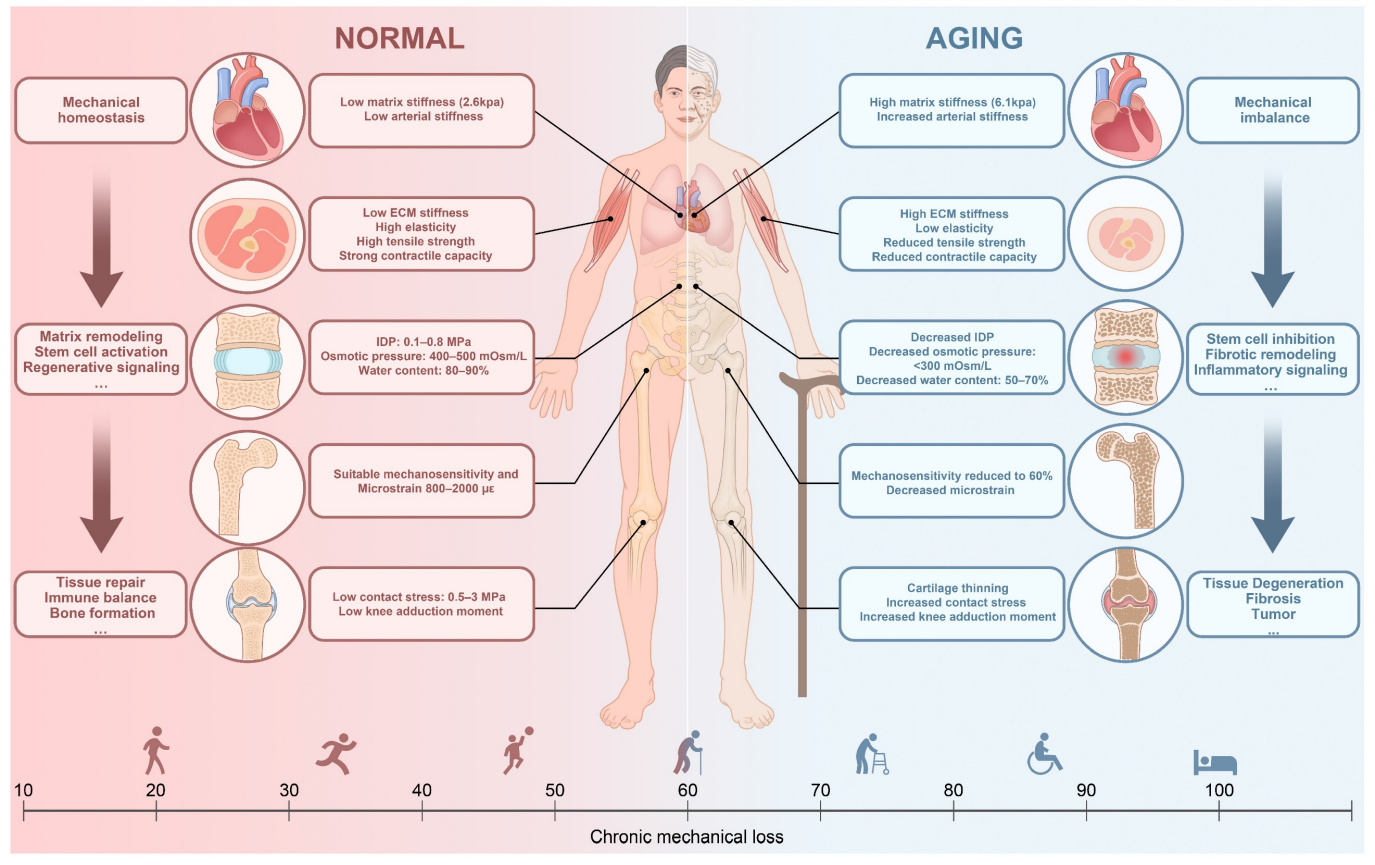
Systemic mechanical remodeling across lifespan shapes tissue homeostasis and degeneration. This schematic summarizes age-associated alterations in mechanical properties across major organs and musculoskeletal tissues. In youth, balanced mechanical loading supports matrix remodeling, stem-cell activation, mechanosensitive signaling, and coordinated tissue repair. With aging, increased stiffness, impaired force-transfer, reduced mechanosensitivity, and altered osmotic and strain environments disrupt mechano-immune homeostasis, leading to inflammation, fibrosis, degeneration, and diminished regenerative capacity. Together, chronic mechanical loss drives the transition from mechanical homeostasis to mechanical imbalance across the lifespan.

In young individuals (Figure 1, left), mechanical homeostasis is maintained across multiple organ systems. Key tissues—including the heart, skeletal muscle, intervertebral discs (IVD), and bone—are characterized by relatively low stiffness, high elasticity, and efficient force transmission 9-12. Under these conditions, physiological loading is translated into coordinated mechano-immune signaling that activates resident tissue cells and immune cells, promotes extracellular matrix (ECM) remodeling, and sustains regenerative pathways across tissues 13, 14. In bone, appropriate microstrain stimulates the periosteum, tissue-resident macrophages, and osteoblasts, thereby supporting continuous bone remodeling.

With aging (Figure 1, right), progressive mechanical remodeling occurs across organs and tissues. Increased in myocardial stiffness 15, heightened skeletal muscle rigidity accompanied by reduced elasticity and contractile capacity 16, 17, and a gradual decline in the efficiency of mechanical conduction in bone 18, 19 represent typical examples. These alterations disrupt the tightly regulated mechano-immune balance. At the cellular level, both innate and adaptive immune populations exhibit altered responsiveness to mechanical stimuli, with phenotypic shifts toward pro-inflammatory states that accelerate age-related pathology 20-22. Concurrently, structural changes in the aging ECM reshape the mechanical microenvironment of immune cells, subjecting these cells to persistent aberrant mechanical stimuli 23, 24. This age-related disruption of mechano-immune regulation contributes to bone loss, joint degeneration, annulus fibrosus rupture in IVD, and inflammation-associated sarcopenia, ultimately reinforcing a self-perpetuating cycle of degenerative musculoskeletal diseases.

Systematic elucidation of mechano-immune imbalance in the context of aging is crucial for clarifying the mechanisms underlying degenerative musculoskeletal diseases. A mechanistic framework centered on disrupted mechano-immune coupling provides deeper insight into disease initiation and progression. More importantly, this perspective informs potential clinical strategies. Integration of controlled mechanical loading—such as exercise, traction, or targeted biomechanical stimulation—with modulation of the immune microenvironment, including regulation of macrophage polarization and immunometabolic reprogramming, together with tissue engineering and regenerative medicine approaches, may facilitate restoration or reconstruction of mechano-immune coupling during the early stages of disease 25-27.

## 1. Mechano-Immunity in the Skeletal Muscle System

### 1.1 Mechanical Transduction in Immune Cells

**Figure 2:**
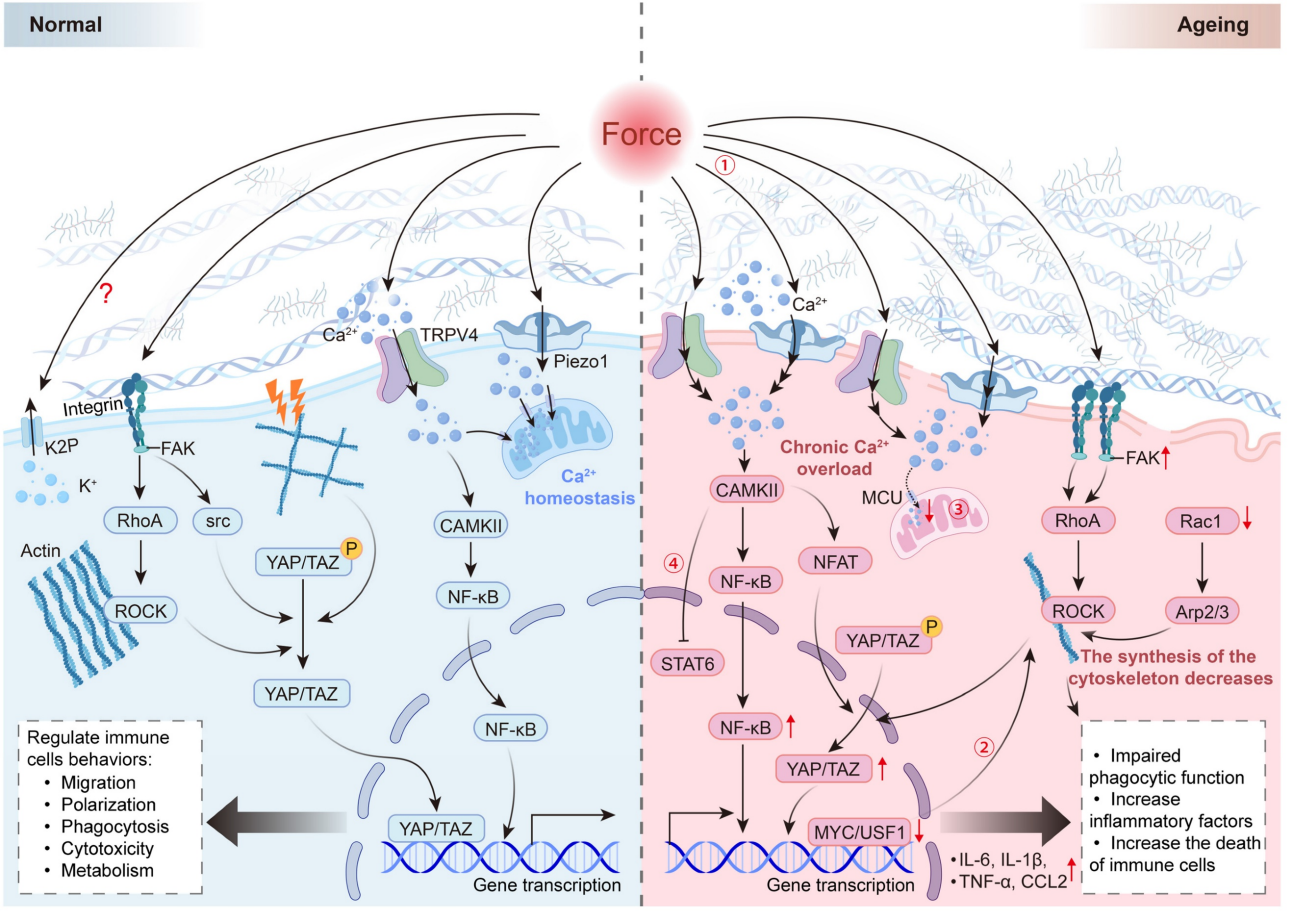
Schematic illustration of mechanotransduction cascades in normal and ageing immune cells. In normal immune cells, mechanical cues—including ECM stiffness, tension, compression, and shear stress—are sensed by mechanosensors such as integrins, Piezo1, and TRPV4. These inputs induce transient and well-controlled Ca²⁺ influx, cytoskeletal remodeling, and YAP/TAZ nuclear translocation, thereby maintaining NF-κB/STAT signaling homeostasis and supporting migration, phagocytosis, polarization, and metabolic activity. In contrast, aged immune cells exposed to stiffened ECM exhibit altered mechanosensing, leading to chronic Ca²⁺ overload, reduced mitochondrial buffering capacity, and aberrant activation of the ROCK-CAMKII-NF-κB/NFAT axis. These defects, together with diminished cytoskeletal synthesis and dysregulated YAP/TAZ activity, result in elevated inflammatory cytokines, impaired phagocytosis, and increased cell death, ultimately driving mechano-immune imbalance (①-④).

Immune cells continuously perceive mechanical cues within the surrounding microenvironment, including ECM stiffness, compression, stretch, and shear stress (Figure 2, left; Table 1) 28-30. Mechanotransduction is initiated at the plasma membrane through mechanosensitive structures such as integrins, T cell receptor (TCR), B cell receptor (BCR), piezo-type mechanosensitive ion channel component 1 (Piezo1), transient receptor potential vanilloid 4 (TRPV4), and two-pore-domain potassium (K2P) channels, including TREK-1/2 and TRAAK 31-37. K2P channels are enriched in innate immune cells, particularly macrophages, where regulation of K^+^ flux contributes to modulation of inflammatory responses 38, 39. Intrinsic mechanosensitivity positions these channels as potential mediators of macrophage responses to mechanical cues, although precise roles in macrophage mechanotransduction remain to be fully defined 38, 39.

Activation of mechanosensors initiates two interelated signaling axes: biochemical cascades and cytoskeletal force transmission. Mechanical clustering of integrins promotes recruitment of focal adhesion kinase (FAK), Src, and phospholipase C (PLC), leading to activation of mitogen-activated protein kinase (MAPK) and nuclear factor kappa-light-chain-enhancer of activated B cells (NF-κB) signaling pathways 40, 41. Concurrently, Ca²^+^ influx through Piezo1 and TRPV4 stimulates calmodulin-dependent kinase II (CaMKII) and calcineurin, facilitating nuclear translocation of nuclear factor of activated T cells (NFAT) and NF-κB 42.

In parallel, mechanical tension is propagated along the actin-microtubule cytoskeleton to the nucleus via the linker of nucleoskeleton and cytoskeleton (LINC) complex and lamins A/C, thereby modulating nuclear mechanics and chromatin organization 43-45. Mechanosensitive transcriptional regulators, including yes-associated protein (YAP), transcriptional coactivator with PDZ-binding motif (TAZ), and NF-κB, integrate these inputs to reshape transcriptional programs^46^, ^47^.

Collectively, these coordinated pathways regulate immune cell migration, differentiation, metabolic adaptation, and effector function. Under physiological mechanical conditions, such mechanisms support immune homeostasis, whereas persistent mechanical stress may amplify inflammatory responses and contribute to tissue dysfunction.

### 1.2 Mechano-Immunity in Musculoskeletal Homeostasis

#### 1.2.1 Bone Homeostasis

Bone tissue serves not only as a core site of mechanical force transmission and metabolic activity but also as an essential niche for immune cell residence. Immune cell populations are regionally distributed within the periosteum and bone marrow, where distinct mechano-immune regulatory roles contribute to the maintenance of skeletal homeostasis (Table 2) 48, 49.

The periosteum, forming the outer layer of bone, represents a highly metabolically active and mechanosensitive compartment enriched with macrophages and regulatory T cells (Tregs) 50, 51. Mechanical loading has been shown to activate Piezo1 in CD68^+^F4/80^-^ myeloid cells within the periosteum, driving differentiation into CD68^+^F4/80^+^ macrophages. These macrophages secrete transforming growth factor-β (TGF-β), facilitating recruitment of osteoprogenitor cells to the periosteal surface and promoting cortical bone deposition 52. Periostin has emerged as a mechanoresponsive mediator linking mechanical stimuli to immune regulation during bone repair. Mechanical stimulation upregulates periostin expression in macrophages, promotes M2 polarization, and increases TGF-β secretion, thereby enhancing osteogenesis and angiogenesis within the periosteal microenvironment 53-55.

Within bone tissue, the marrow harbors diverse immune cell populations that actively participate in the regulation of skeletal homeostasis 56, 57. Mechanical loading induces Piezo1-dependent Ca²⁺ influx and modulates p53 acetylation, promoting polarization of bone marrow macrophages (BMMs) toward an M2 phenotype. These macrophages secrete osteogenic and angiogenic mediators, including TGF-β1, vascular endothelial growth factor A (VEGFA), and bone morphogenetic protein 2 (BMP2), thereby stimulating differentiation of bone marrow stromal cells (BMSCs) 58, 59. Mechanical cues further coordinate metabolic support for osteogenesis. Loading enhances BMM-derived reticulocalbin-2 (RCN2) expression, activating Neuropilin-2/Integrin β1-cyclic adenosine monophosphate-protein kinase A (cAMP-PKA) signaling to promote lipolysis in marrow adipocytes and provide metabolic substrates for BMSC-driven osteogenesis and hematopoietic activity 60, 61. In parallel, mechanically stimulated BMMs release ubiquitin C-terminal hydrolase L3 (UCHL3)-containing exosomes that activate Smad1 signaling in BMSCs 62, 63, and transfer mitochondria via extracellular vesicles to enhance cellular metabolic capacity 64. In cortical bone, physiological loading enhances osteocyte autophagy through mechanistic target of rapamycin complex 2 (mTORC2), increasing adenosine triphosphate (ATP) production and osteogenic signaling while suppressing osteoclast activity through regulation of colony-stimulating factor and receptor activator of NF-κB ligand/osteoprotegerin (RANKL/OPG) balance 65.

Beyond BMMs, additional immune cell subsets contribute to mechano-immune regulation in bone. γδT cells rapidly secrete interleukin-17A (IL-17A) during orthodontic tooth movement, stimulating fibroblasts to express RANKL and promote osteoclast activation. IL-17A also recruits and activates monocytes and neutrophils, thereby accelerating bone remodeling 66. Conversely, mechanical loading induces c-Jun^+^ neutrophils to secrete oncostatin M, supporting bone regeneration 67.

Functional lymphatic vessels have recently been identified within bone tissue, where regulation of the immune microenvironment occurs through interstitial fluid drainage processes closely linked to mechanical stimulation 68, 69.

#### 1.2.2 Joints

Within the joint, immune cells are predominantly localized in the synovial tissue. The intimal layer of the synovium consists of two major cell types: type A synoviocytes (macrophage-like) and type B synoviocytes (fibroblast-like). Type A synovial macrophages form a lining along the synovial cavity, establishing a semi-permeable immunological barrier responsible for recognition and clearance of cellular debris, apoptotic cells, and damage-associated molecular patterns (DAMPs) from synovial fluid. This barrier function restricts excessive infiltration of circulating immune cells and contributes to maintenance of immune homeostasis within the joint cavity 70-72.

Moderate mechanical stimulation represents a crucial regulatory factor in preserving this homeostatic state (Table 2). During early OA, mechanical-immune negative feedback mechanisms may attenuate disease progression. Appropriate mechanical loading has been shown to inhibit activation of the phosphatidylinositol 3-kinase (PI3K)/protein kinase B (AKT)/NF-κB signaling pathway in synovial macrophages, thereby limiting M1 polarization and reducing production of pro-inflammatory cytokines, such as interleukin-1 (IL-1) and tumor necrosis factor-α (TNF-α) 73. Regular mechanical stimulation or exercise also elevates local and systemic levels of lipoxin A4. Under the synergistic action of mechanical cues and lipoxin A4 signaling, synovial macrophages shift toward M2 phenotype, characterized by secretion of anti-inflammatory cytokines such as interleukin-10 (IL-10) and TGF-β. These factors inhibit chondrocyte pyroptosis, promote tissue repair, and reinforce joint homeostasis 74. Moreover, mechanical loading suppresses osteoclast activity in subchondral bone through upregulation of Wingless-type MMTV integration site family member 3A (Wnt3a), thereby reducing bone resorption and structural deterioration and preserving overall joint integrity 75.

#### 1.2.3 Muscles

Under homeostatic conditions, skeletal muscle contains a relatively sparse yet functionally diverse population of resident immune cells, primarily tissue-resident macrophages with smaller subsets of T lymphocytes. These immune populations contribute to metabolic regulation, maintenance of satellite-cell quiescence and activation, as well as routine tissue turnover and repair 76, 77.

Mechanical forces serve as central upstream regulators of this immune milieu (Table 2). During acute muscle injury, physiological loading promotes interstitial fluid dynamics and facilitates clearance of pro-inflammatory cytokines, thereby limiting neutrophil influx and reducing early inflammatory mediators such as matrix metalloproteinase-9 (MMP-9) and C-C motif chemokine ligand 3 (CCL3) 78. As the inflammatory phase resolves, sustained mechanical activity drives macrophage polarization toward an M2-like reparative phenotype, enhancing interleukin-4 (IL-4)-dependent regeneration, suppressing interleukin-6 (IL-6)-driven inflammation, and facilitating satellite-cell expansion and myofiber hypertrophy 79, 80. Over longer durations, exercise-induced mechanical signaling expands an interleukin-6 receptor α (IL-6Rα)-dependent intramuscular regulatory T cell population that contributes to muscle regeneration and functional performance 81.

In addition to classical inflammation-regeneration dynamics, skeletal muscle contains a specialized neural-muscular-immune interface within muscle spindles. Spindle-associated macrophages metabolize myofiber-derived glutamine into glutamate during contraction or stretch, thereby modulating sensory afferent discharge. Depletion of these macrophages disrupts proprioceptive feedback and motor coordination, showing that immune cells act as metabolic intermediaries of mechanical signals and key regulators of neuromuscular homeostasis 82.

#### 1.2.4 Tendons and Enthesis

The enthesis functions as the transitional interface between tendon and bone and is composed of four distinct zones—tendon, unmineralized fibrocartilage, mineralized fibrocartilage, and bone—enabling efficient transfer of tensile and compressive forces across tissues 83. Within tendons, CD206⁺ M2-like resident macrophages are present in the inner connective tissue layer from the embryonic stage. Spatial maintenance of these macrophages is supported by colony-stimulating factor 1 derived from fibroblasts, underscoring an essential role in tendon development and homeostasis 84. Following tendon injury, M1 macrophages predominate during the early inflammatory phase to facilitate clearance of necrotic tissue. Subsequent transition toward an M2 phenotype supports matrix remodeling through secretion of growth factors , thereby promoting tissue repair and limiting adhesion formation 85.

Mechanical loading represents a pivotal upstream regulator of immune homeostasis and dynamic ECM renewal in tendon tissue (Table 2). Under physiological tensile conditions, shifts in local cytokine profiles favor macrophage polarization toward an M2-like reparative phenotype, promoting secretion of anti-inflammatory and pro-regenerative mediators. Such responses facilitate coordinated ECM turnover and proper collagen fiber organization 86. In contrast, excessive or aberrant mechanical loading sustains M1 macrophage activation, leading to disorganized ECM remodeling and chronic inflammatory states 87. Similar patterns of mechanical-immune coupling are likely present at the enthesis; however, current evidence remains limited and does not yet permit definitive characterization of these mechanisms.

#### 1.2.5 Intervertebral Discs

In contrast to synovial joints, IVD contain minimal immune cell infiltration under physiological conditions. The cartilaginous endplate and annulus fibrosus together establish a structural barrier that confers relative immune privilege, restricting penetration of circulating immune cells into the nucleus pulposus (NP) compartment 88, 89. The chronically hypoxic and poorly vascularized microenvironment of the disc further constrains immune cell entry and activation 88, 89. Available evidence indicates that only sparse macrophage-like or mononuclear-like cells are occasionally detected in the outer annulus fibrosus or adjacent endplate regions, where limited roles in local immune surveillance and ECM homeostasis have been proposed 90.

## 2. Aging-Related Mechanical-Immune Imbalance

### 2.1 ECM Aging Shapes the Mechano-Immune Niche

In healthy tissues, the ECM provides a compliant and structurally organized scaffold that supports normal cellular function. Aging is accompanied by lifelong exposure to subtle mechanical strain, metabolic perturbation, and oxidative microinjury. Repair processes are frequently incomplete, and cumulative microdamage progressively reshapes ECM architecture over decades. Collagen deposition increases, lysyl oxidase-mediated cross-linking intensifies, elastin undergoes fragmentation, and levels of proteoglycans and hyaluronic acid decline, while advanced glycation end products accumulate. These alternations collectively enhance matrix stiffness and tensile resistance, characteristic features of aged ECM. Elevated stiffness disrupts mechanotransduction in epithelial, endothelial, stromal, and immune cells, contributing to chronic low-grade inflammation and impaired regenerative capacity. Progressive accumulation of microdamage followed by maladaptive ECM remodeling represents a central biomechanical pathway in age-related tissue dysfunction 24, 91-93.

Within innate immune populations, particularly macrophages, ECM stiffening enhances integrin engagement and activation of mechanosensitive ion channels, converging on Rho-associated coiled-coil containing protein kinase (ROCK), es-associated protein/transcriptional coactivator with PDZ-binding motif (YAP/TAZ), signal transducer and activator of transcription 6 (STAT6), and NF-κB signaling pathways. 23, 94-97. Sustained nuclear signaling alters chromatin accessibility and stabilized a pro-inflammatory transcriptional program characterized by increased production of IL-1, TNF-α, interferon-γ (IFN-γ), and TGF-β production. These mediators promote fibro-inflammatory activation and further matrix remodeling. 23, 98, 99. Persistent inflammatory signaling also induces metabolic stress and cell death, resulting in release of DAMPs that amplify sterile inflammation. Through these interconnected mechanisms, ECM stiffening established a self-perpetuating mechanical-immune feedback loop that reinforces tissue degeneration 100-103.

Within adaptive immunity, increased matrix stiffness and architectural disorganization impair tissue surveillance and migratory capacity. Tissue-resident memory T (TRM) cells rely on CD103 and CD49a-mediated adhesion, CD69-dependent retention, and nuclear mechanosensing mechanisms that enable stepwise interstitial migration 104-108. Age-associated ECM remodeling disrupts these processes, resulting in TRM mislocalization and functional exhaustion. Compromised positioning and reduced effector competence weaken surveillance against latent viral infection and transformed cells, thereby contributing to persistent low-grade inflammation 109-111. Excessive ECM stiffness exceeding approximately 40 kPa has been shown to induce CD8^+^ T cell exhaustion through activation of the Piezo1-calmodulin-dependent kinase II (CaMKII) axis, leading to diminished IFN-γ and perforin secretion. Concurrently, GRHL3-RNF114-F-actin signaling reduces immune synapse tension, further limiting cytotoxic efficacy 112, 113.

Comparable mechanical-immune dysregulation is observed in degenerative musculoskeletal disorders. In OA, matrix stiffening potentiates macrophage-mediated synovial inflammation and fibrotic remodeling 94, 114-116. In IDD, transient stiffening followed by matrix breakdown disrupts force transmission and perpetuates aseptic inflammation 117-119. In OP, ECM degradation and trabecular deterioration heighten skeletal susceptibility to mechanical unloading, further destabilizing bone immune homeostasis (Figure 2, right) 120, 121.

### 2.2 Mechanobiological Dysfunction in Aging Immune Cells

Aging is accompanied by impaired mechanotransduction in tissue-resident matrix-producing and contractile cells, including cardiomyocytes, smooth muscle cells, and muscle stem cells. Attenuation of YAP/TAZ signaling has been linked to aberrant activation of the cyclic GMP-AMP synthase-stimulator of interferon genes (cGAS-STING) pathway, accelerating ECM senescence and accumulation of apoptotic and senescent cells. These alterations foster a pro-inflammatory tissue microenvironment 122-124. Immune cells, despite rapid turnover relative to many somatic cell types, also exhibit age-associated functional decline. Progressive accumulation of senescent immune populations contributes to dysregulated chronic inflammation and tissue fibrosis 125-127. In addition to established metabolic and transcriptional reprogramming, emerging evidence highlights a less explored dimension of immunosenescence characterized by defects in mechanical sensing and force transduction. Disruption of mechanobiological responsiveness may represent a critical but underrecognized contributor to age-related immune dysfunction.

Accumulating evidence indicates that aging immune cells exhibit broad impairments in mechanosensing, cytoskeletal remodeling, and force execution. In aged macrophages, defective Rac1/Arp2/3-dependent F-actin polymerization, together with reduced expression of transcription factors such as MYC and upstream stimulatory factor 1 (USF1), compromises cytoskeletal integrity, adhesion capacity, and ECM remodeling. These alterations translate into reduced migration, phagocytosis, and efferocytosis 21, 22, 128. Concurrent downregulation of mitochondrial calcium uniporter components, including MCU and MICU1, reduces mitochondrial Ca²^+^ buffering and disrupts cytosolic Ca²^+^ oscillatory patterns. Altered calcium homeostasis may heighten cellular sensitivity to mechanical stimuli, such that physiological levels of matrix stiffness or tissue tension trigger excessive Ca²⁺ influx through Piezo1 and TRPV4 channels, thereby aggravating inflammatory responses and tissue injury. Hyperglycemic conditions further amplify these effects 20, 129, 130. Despite these observations, current research remains largely descriptive and lack systematic characterization of mechanical sensing properties and mechanotransductive responses in aged macrophages.

Comparable defects are evident in adaptive immune populations. Aged T cells exhibit disrupted F-actin dynamics and impaired nucleo-cytoskeletal coupling, leading to weakened immunological synapse force generation, reduced migratory efficiency, and elevated thresholds for TCR activation, ultimately diminishing cytotoxic and helper functions 131, 132. Senescent dendritic cells demonstrate attenuated C-C motif chemokine receptor 7 (CCR7) signaling and defective cytoskeletal remodeling, limiting effective navigation through ECMs, homing to lymph nodes, and antigen presentation capacity 133, 134.

Collectively, these findings highlight that immunosenescence encompasses not only alterations in molecular signaling networks and metabolic pathways but also fundamental disruptions in mechanical sensing and force transduction. Such mechanobiological deficits may constitute a critical and underrecognized dimension of age-related immune dysfunction.

## 3. Mechanical-Immune Imbalance in Degenerative Musculoskeletal Diseases

### 3.1 Osteoporosis

**Figure 3:**
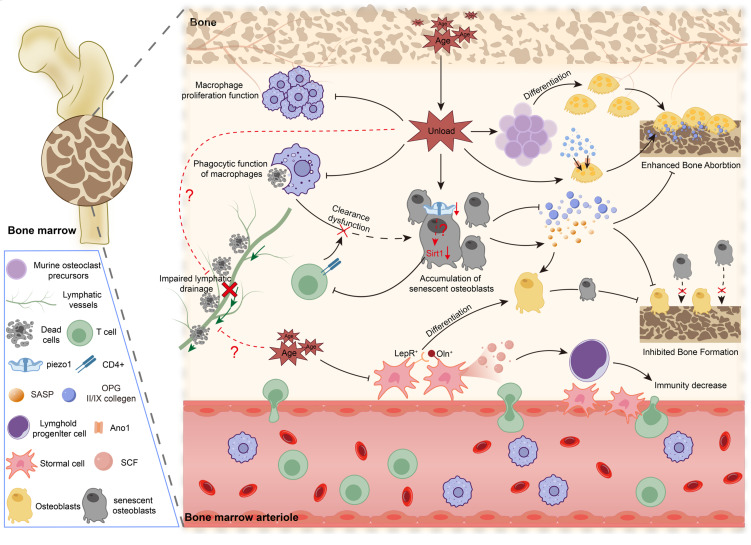
Mechanical unloading driven immune dysregulation in osteoporosis. Ageing-associated mechanical unloading disrupts bone-immune homeostasis by impairing macrophage proliferation and phagocytic clearance, promoting the accumulation of senescent osteoblasts, and enhancing osteoclast differentiation and bone resorption. Concurrently, lymphatic drainage may be further reduced under mechanical unloading and ageing, exacerbating inflammatory and metabolic stress within the bone marrow niche. These changes collectively diminish osteogenic potential, accelerate bone loss, and contribute to compromised immunity, underscoring the pivotal role of mechano-immune dysfunction in age-related osteoporosis.

Osteoporosis represents one of the most prevalent age-associated disorders affecting the elderly population 1. Age-related skeletal deterioration reflects the convergence of two major mechanical deficits 135, 136. Reduced mobility and physical activity diminish habitual mechanical loading, thereby suppressing osteoblastic bone formation and favoring osteoclastic resorption. In parallel, skeletal mechanosensation depends on osteocytes and the lacunocanalicular system (LCS), a specialized structural and fluidic network that transmits mechanical stimuli and coordinates mechano-immune signaling between the bone matrix and bone marrow. Advancing age is associated with quantitative reduction and structural degeneration of the LCS, leading to impaired mechanical signal propagation and a marked reduce in osteocyte-experienced fluid shear stress, which declines to approximately one-third of levels observed in young bone 18, 19. Such mechanical insufficiency effectively produces a state of functional unloading for osteoblasts, osteoclasts, and marrow-resident immune cells. The combined effects of diminished external mechanical stimulation and intrinsic defects in mechanotransduction disrupt mechanical-immune homeostasis, driving chronic low-grade inflammation and progressive skeletal degeneration (Figure 3) 120, 121, 136.

#### 3.1.1 Cellular Mechanosensory Dysfunction in the Aging Skeleton

In addition to degeneration of the LCS, aging compromises mechanosensing within stromal and osteolineage compartments, thereby destabilizing the immuno-bone axis. LepR^+^Oln^+^ osteogenic progenitors, short-lived and highly mechanoresponsive stromal cells, decline with advancing age and reduced physical activity. Loss of this population reduces osteogenic output and lymphopoietic support. Restoration through exercise in a Piezo1-dependent manner underscores a central role as a mechanical hub integrating skeletal and immune homeostasis 27. Conversely, aging is associated with expansion of a LepR^+^Sca1^+^Cxcl9^+^ stromal subset enriched for interferon-response signatures. These cells form inflammatory perivascular niches that suppress lymphoid progenitor development and may further impair the mechanical responsiveness in adjacent osteogenic stromal populations 137.

Mechanosensory defects also extend to mature osteocytes. Reduced expression of Piezo1 in aged osteocytes, with declines of approximately 30-50%, is associated with a 40% reduction in OPG, thereby diminishing inhibitory control over osteoclast activity 138. Similarly, decreased Piezo1 expression in osteoblasts results in type II and IX collagen deficiency, weakening integrin-mediated inhibitory signaling toward osteoclasts and increasing bone resorptive activity by approximately 2.1-fold 139. Collectively, these alterations reflect progressive deterioration of cellular mechanosensing capacity that contributes to age-related skeletal fragility.

#### 3.1.2 Mechanical Unloading Intensifies Immuno-Skeletal Imbalance

Senescent osteoblasts contribute substantially to the progression of osteoporosis. With advancing age, increased secretion of senescence-associated secretory phenotype (SASP) factors, including IL6, interleukin-1β (IL-1β), and MMPs, promotes osteoclastic bone resorption and induces adipogenic differentiation of BMSCs via paracrine mechanisms 140, 141. Under physiological conditions, macrophages and T lymphocytes participate in clearance of senescent osteoblasts; however, immunosenescence compromises this surveillance system 142, 143. Aging-associated downregulation of sirtuin 6 (SIRT6) in macrophages and apoptotic osteoblasts, results in CD47 overexpression and impaired efferocytosis 144, 145. Concurrent reduction of sirtuin 1 (SIRT1) expression in osteoblasts diminishes chemokine production and CD4^+^ T cell recruitment, further weakening immune-mediated clearance 146. Furthermore, senescent M1 macrophages and neutrophils secrete elevated levels of grancalcin and microRNA-enriched extracellular vesicles, which enhance adipogenic differentiation of BMSCs and propagate senescence-associated changes across multiple tissues and organs 147, 148.

In older individuals, reduced physical activity combined with degeneration of the LCS further exacerbates immunosenescence, accelerating chronic medullary inflammation and progressive bone loss. Evidence from microgravity models demonstrates that mechanical unloading impairs maturation of BMMs and disrupts cytoskeletal organization, resulting in reduced proliferation and significantly suppressed M1/M2 polarization capacity 149-151. Mechanistic analyses indicate suppression of RAS/ERK/NF-κB signaling pathways in macrophages, thereby limiting proliferative and polarization responses, while concurrent activation of p53 signaling induces cell cycle arrest 150. Observations from spaceflight studies further reveal markedly reduced phagocytic activity of monocyte-derived macrophages and neutrophils following return to Earth 152-154, accompanied by features consistent with immunosenescence such as functional exhaustion and increased production of pro-inflammatory cytokines 155. Mechanical unloading also profoundly reduces SIRT1 expression in osteoblasts, likely secondary to diminished activation of the Piezo1-CaMKII-CREB axis 156, 157. Reduced SIRT1 expression compromises chemokine-mediated recruitment of CD4^+^ T cells and impairs immune-mediated clearance of apoptotic or senescent osteoblasts 146, 158, 159. Inefficient clearance promotes accumulation of dysfunctional cells within the bone marrow niche and sustains a chronic pro-inflammatory microenvironment. Collectively, these alterations establish a persistent inflammatory state closely resembling the medullary inflammation characteristic of age-related osteoporosis.

Conversely, absence of mechanical loading directly accelerates bone resorption 160. Lack of mechanical stimulation increases the opening frequency of the anoctamin 1 (Ano1) channel in osteoclasts by approximately threefold 161, thereby enhancing resorptive activity through activation of the polycystin-1 (PC1)-TAZ signaling axis 162. Mechanical deprivation also shifts hematopoietic lineage commitment toward osteoclast differentiation, further amplifying bone resorption 163. Under conditions of extreme unloading, such as space microgravity, differentiation of osteoclast precursors is markedly increased, leading to heightened bone resorptive activity and accelerated skeletal calcium loss 164, 165.

#### 3.1.3 Potential Skeletal Lymphatic Remodeling in Aging and Mechanical Unloading

Recent investigations have identified functional lymphatic vessels within bone tissue, challenging the longstanding view that the skeletal system lacks a lymphatic network. These vessels participate in interstitial fluid drainage, immune cell trafficking, and coordination of bone remodeling processes 166. Despite these advances, adaptive responses of skeletal lymphatics to systemic aging and altered mechanical loading conditions remain insufficiently defined.

Aging is associated with reduced lymphangiogenesis and diminished sensitivity to vascular endothelial growth factor C (VEGF-C)/vascular endothelial growth factor receptor 3 (VEGFR3) signaling. Lymphatic vessels within bone similarly exhibit functional decline during aging, with potential consequences for osteoblast maintenance and immune cell dynamics 166-168. In the aged bone microenvironment, accumulation of apoptotic osteocytes and impaired immune clearance have been documented. Decreased lymphatic density and function may exacerbate this condition by limiting cellular debris and inflammatory mediators, thereby sustaining a chronic inflammatory milieu and further disrupting skeletal homeostasis 144-146.

Mechanical cues function as critical regulators of lymphatic morphogenesis and maintenance. Fluid shear stress affects lymphatic endothelial cell morphology, arrangement, migration, and cell cycle progression, thereby regulating vessel dilation, structural remodeling, and functional capacity 68. During progression of osteoporosis, mechanical unloading and ECM degradation may deprive biomechanical support for skeletal lymphatics, compromising structural stability and flow-dependent signaling. Experimental evidence indicates that chronic mechanical unloading, including limb immobilization or microgravity exposure, reduces lymphatic pump activity, drainage efficiency, and vessel density 169-171.

In age-related osteoporosis, skeletal lymphatics are therefore likely subjected to combined effects of systemic aging and reduced mechanical stimulation, predisposing to lymphatic stasis. Such maladaptive remodeling may favor accumulation of senescent cell, amplify chronic inflammatory bone loss, and further compromise skeletal homeostasis.

Taken together, these considerations position skeletal lymphatic remodeling as an underexplored but potentially critical axis linking aging, mechanical forces, and skeletal integrity.

### 3.2 Osteoarthritis

**Figure 4:**
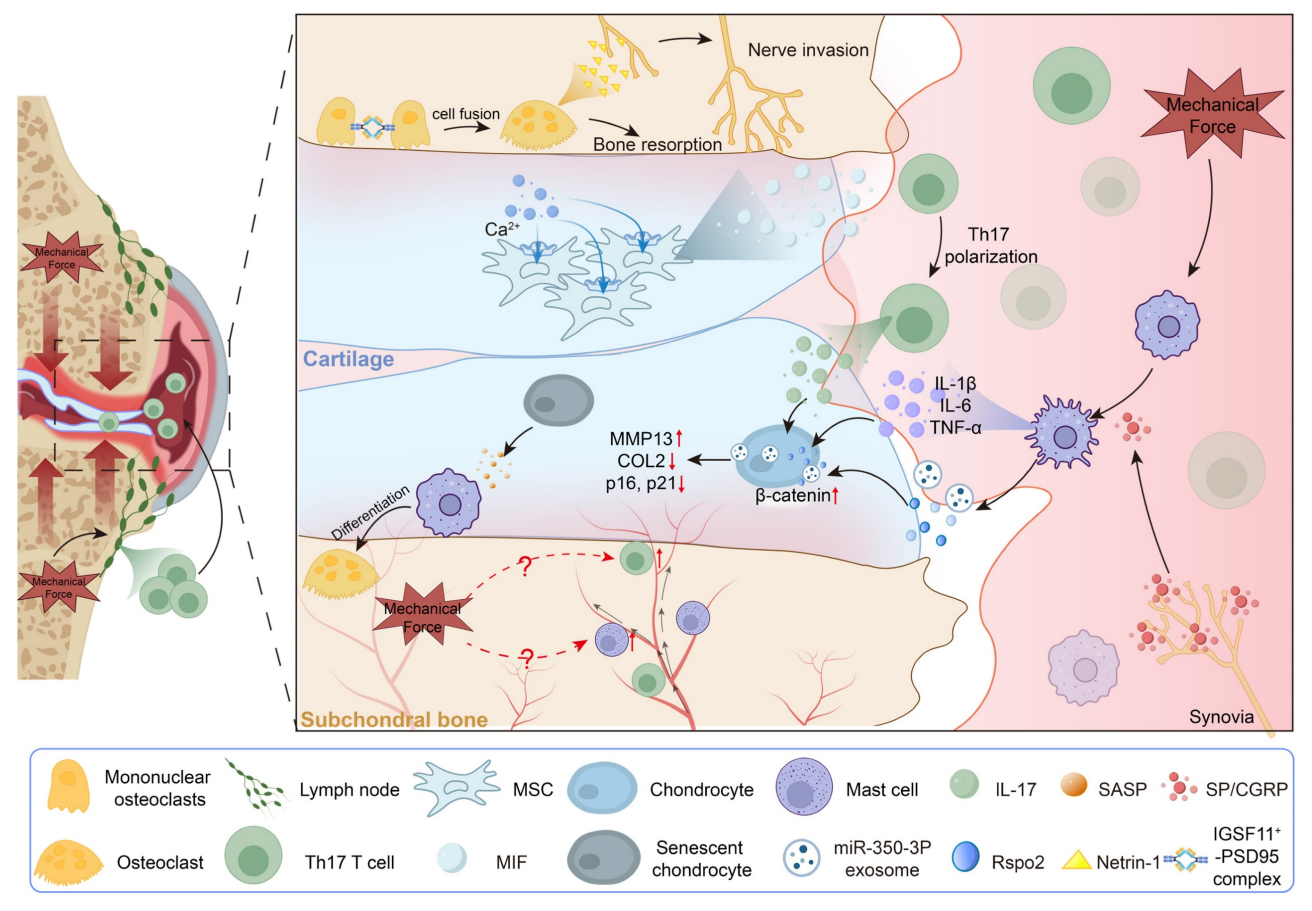
Mechanical loading drives immune-mediated synovial, cartilage and subchondral bone pathology. Mechanical loading promotes T-cell egress from lymph nodes and their subsequent recruitment into the synovium, where it drives macrophage M1 polarization and the production of IL-1β, IL-6, TNF-α, miRNAs, and Rspo2. This macrophage response is further amplified by SP/CGRP⁺ sensory afferents, reinforcing catabolic signaling to chondrocytes and inducing β-catenin activation, MMP13 upregulation, COL2 loss. Mechanical cues also stimulate cartilage MSCs to release MIF, promoting TH17 differentiation. Within the subchondral bone, loading triggers osteocyte fusion and netrin-1 production, facilitating sensory nerve ingrowth, while SASP factors from senescent chondrocytes enhance osteoclastogenesis and bone resorption.

OA, the most prevalent form of degenerative joint disease and a leading contributor to global disability, affects more than 500 million individuals worldwide, with incidence rising sharply after 50 years of age 172, 173. Aging is accompanied by progressive structural deterioration of joint components, particularly within the knee, including degeneration of articular cartilage and meniscal tissue. These changes are associated with increased joint contact stress, reduced skeletal muscle strength, and elevated knee varus moment, collectively contributing to mechanical imbalance 172-175. Biomechanical alterations weaken the load-bearing and shock-absorbing properties of cartilage, leading to localized stress concentration and compromised force redistribution across the joint surface 176, 177. Muscle atrophy, particularly involving the quadriceps and hamstrings, further destabilizes joint mechanics and amplifies medial compartment loading, thereby accelerating cartilage degeneration and subchondral bone sclerosis 178, 179. At the cellular level, senescent chondrocytes exhibit impaired mechanosensory capacity, while dysregulated osteoclast-osteoblast coupling promotes ECM degradation and microarchitectural damage. These interrelated processes establish a self-reinforcing cycle characterized by stress concentration, microdamage accumulation, tissue dysfunction, and progressive mechanical overload, ultimately driving OA progression (Figure 4) 180.

#### 3.2.1 Pathways of Immune Cell Infiltration in Osteoarthritis

Histologically, synovitis in OA is characterized by synovial lining hyperplasia, sublining fibrosis, and pronounced neovascularization. In response to locally elevated cytokines and adhesion molecules, leukocytes migrate from the vascular lumen into the synovial stroma. Macrophages and T lymphocytes constitute the predominant immune populations, whereas mast cells, B cells, and plasma cells are present at lower frequencies 70, 181, 182. Persistent joint inflammation disrupts the balance of regulatory macrophage subsets, reduces phagocytic and anti-inflammatory capacity, and induces features of macrophage senescence in periarticular muscle tissue, thereby contributing to muscle atrophy and compromised joint stability 183, 184.

With disease progression, cartilage degradation and subchondral bone remodeling enable vascular invasion across the osteochondral junction, creating additional pathways for immune cell infiltration and dissemination of inflammatory mediators 182, 185. Aging further intensifies this process. Senescent chondrocytes secrete SASP factors, such as IL-6, TNF-α, MMPs, and VEGF. These mediators facilitate neovascularization and promote upward vascular extension from subchondral bone into cartilage tissue 186-188, sustaining a chronic inflammatory milieu that disrupts synovial and adipose tissue homeostasis 189, 190. Infiltration of pro-inflammatory macrophages, activated T lymphocytes, and angiogenic endothelial cells into the cartilage-bone interface establishes a self-perpetuating inflammatory circuit, accelerating cartilage erosion, vascular penetration, and nociceptive sensitization.

#### 3.2.2 Mechanical Dysregulation Fuels Osteoarthritis Inflammation

During OA progression, infiltrating immune cells operate within a mechanically altered joint environment, generating a distinct mechano-immune pathogenic axis. Persistent mechanical imbalance activates pro-inflammatory signaling pathways that enhance immune cell recruitment and mediator release. Mechanical overload upregulates rap guanine nucleotide exchange factor 3 (Rapgef3) and activates the p65-NF-κB pathway in synovial macrophages, thereby inducing M1 polarization and enhancing secretion of pro-inflammatory cytokines 115. M1-polarized macrophages subsequently release exosomes enriched in miR-350-3p, which are internalized by chondrocytes. These exosomes suppress nuclear receptor binding SET domain protein 1 (NSD1) expression, reduce histone H3 lysine 36 (H3K36) methylation, and accelerate chondrocyte senescence and ECM degradation 191. In age-related OA, aberrant sensory nerve fiber ingrowth increases local release of neuropeptides, such as substance P and α- calcitonin gene-related peptide** (**αCGRP) 192. Mechanical stress enhances macrophage responsiveness to these neuropeptides through downregulation of neurokinin-1 receptor and upregulation of αCGRP receptors, amplifying pro-inflammatory signaling, reinforcing M1 polarization, and intensifying joint inflammation and structural degeneration 193. Mechanical strain also stimulates chondrocytes to secrete the CC chemokine ligand 5 (CCL5), which recruits macrophages and osteoclasts through CC chemokine receptor (CCR)-Akt2 signaling. This process induces subchondral bone loss and establishes a pathological remodeling environment characterized by osteoclast hyperactivation 194.

Abnormal mechanical loading also perturbs adaptive immune responses during OA progression. In murine models of mechanically induced knee OA, T cell populations were significantly increased in regional lymph nodes. Genetic deletion of TCRαβ substantially attenuated bilateral cartilage degeneration and osteophyte formation induced by mechanical stress. In contrast, pharmacological inhibition of T cell efflux from lymph nodes mitigated cartilage degradation without significantly affecting osteophyte development, indicating distinct contributions of T cell subsets and trafficking pathways to structural joint changes 116. Additional studies have revealed that load sensing through Piezo1 in MSCs activates the STAT1-HK2 glycolytic pathway, inducing secretion of macrophage migration inhibitory factor (MIF). This cascade enhances recruitment and polarization of T helper 17 (Th17) cells, thereby amplifying OA-associated inflammation and bone destruction 195. These findings support a model in which mechanical forces not only regulate T cell trafficking into the joint but also modulate intra-articular T-cell function to accelerate OA progression.

Subchondral bone similarly undergoes substantial immune infiltration during OA progression. Vascular invasion is accompanied by increase proportions of CD8⁺ T cells (18.84%), activated mast cells (17.37%), activated CD4⁺ memory T cells (9.12%), and diverse macrophage subsets 196, 197. In other tissues, mast cells are highly responsive to mechanical stimulation, which can activate mechanosensitive channels such as Piezo1 and TRPV4, as well as integrin-mediated signaling pathways, leading to degranulation and release of inflammatory mediators. Alterations in matrix stiffness and cytoskeletal tension further modulate mast cell proliferation, migration, and immune responsiveness 197, 198. However, the specific impact of mechanical forces on mast cell behavior within osteoarthritic joints remains poorly defined and may represent an underrecognized mechanism contributing to disease pathogenesis.

#### 3.2.3 Mechanical Instability Models Expose Osteoarthritis Mechano-Immune Dynamics

Established animal models of mechanically induced OA include anterior cruciate ligament transection, meniscectomy, and destabilization of the medial meniscus (DMM). Each approach generates joint instability and abnormal mechanical loading, leading to progressive osteoarthritic changes 199, 200. Among these models, DMM most closely resembles human primary OA, characterized by gradual progression and a relatively mild phenotype, thereby providing a suitable platform for investigating mechanical-immune coupling. Findings derived from these models collectively illustrate how mechanical imbalance drives synovial inflammation, subchondral bone remodeling, and sensory nerve ingrowth during disease development 200.

Mechanical instability rapidly induces synovial inflammatory responses. Early stages following DMM are marked by synovitis with accumulation of M1-polarized macrophages and elevated expression of IL-1β, IL-6, and TNF-α 201. Mechanical loading further stimulates matrix-degrading enzymes, including ADAMTS4, MMP3, and MMP13, as well as nerve growth factor (NGF), thereby accelerating cartilage degradation 201. Concentrated mechanical stress drives synovial macrophages to release R-spondin-2, which activates β-catenin signaling in chondrocytes and enhances expression of catabolic enzymes, promotes chondrocyte hypertrophy, and facilitates osteophyte formation 202. In aged or chronically overloaded joints, increased cyclin-dependent kinase 8 (CDK8) activity in chondrocytes augments NF-κB/STAT1/3 signaling, induces SASP production, and promotes macrophage transition toward osteoclast-like phenotypes, thereby intensifying inflammation and matrix breakdown 203.

Subchondral bone demonstrates pronounced sensitivity to abnormal mechanical loading, with remodeling alterations frequently preceding overt cartilage degeneration 204. DMM induces expansion of IgSF11⁺ osteoclasts, which enhance osteoclast differentiation and fusion through interactions involving postsynaptic density protein 95, thereby exacerbating bone resorption 205-207. Within two weeks following DMM surgery, activation of the RANKL-UCHL1-sCD13 negative feedback pathway is detectable in osteoclast precursors, serving to restrain excessive resorptive activity during early OA. However, age-associated decline in UCHL1 expression may impair this regulatory loop, potentially accelerating disease progression 208-210. In parallel, monocyte-derived pre-osteoclasts secrete elevated levels of platelet-derived growth factor-BB (PDGF-BB), inducing aberrant angiogenesis and sensory nerve ingrowth within the subchondral compartment 211. Under mechanical stress, osteoclasts upregulate collagen type VI alpha 1 chain (COL6A1) to activate the EPAC/RAP1 pathway, further enhancing osteoclast differentiation and contributing to structural deterioration of subchondral bone 212.

As DMM-induced OA progresses into the chronic phase, coordinated mechanical-neurological-immune interactions drive persistent pain. Approximately eight weeks post-surgery, nociceptive neurons within the dorsal root ganglia (DRG) become primary responders to abnormal mechanical input, accompanied by marked infiltration of macrophages predominantly exhibiting an M1-polarized phenotype. These macrophages release pro-nociceptive mediators, such as IL-1β, TNF, reactive oxygen species (ROS), C-X-C motif chemokine ligand 10 (C-X-C) motif chemokine ligand 10 (CXCL10), and CXCL2, which enhance DRG neuronal excitability and facilitate pain transmission 213-216. Sensory neurons further secrete NGF and fractalkine; fractalkine cleavage by cathepsin S activates spinal microglia, leading to central sensitization that synergizes with peripheral inflammation to maintain chronic hyperalgesia 217, 218. Concurrently, abnormal mechanical loading enhances Netrin-1 secretion from osteoclasts, stimulating aberrant subchondral nerve ingrowth and establishing peripheral nociceptive foci 213.

In aged DMM models, articular cartilage degeneration, subchondral bone remodeling, and osteophyte formation occur earlier and with greater severity than in younger counterparts, suggesting that aging amplifies mechanically driven structural deterioration process induced by mechanical stimuli 219. Although direct comparative data on temporal differences in inflammatory mediator profiles and macrophage polarization between aged and young mice remain limited, aging has been associated with increase propensity toward M1 polarization and reduced capacity for transition to reparative M2 phenotypes. This polarization bias is amplified under conditions of abnormal mechanical stress, accelerating inflammatory progression 220. Consequently, mechanically induced synovitis and pro-inflammatory mediator release in aged individuals are likely to arise earlier and persist longer, thereby accelerating the immunopathological course of OA.

### 3.3. Intervertebral Disc Degeneration

**Figure 5:**
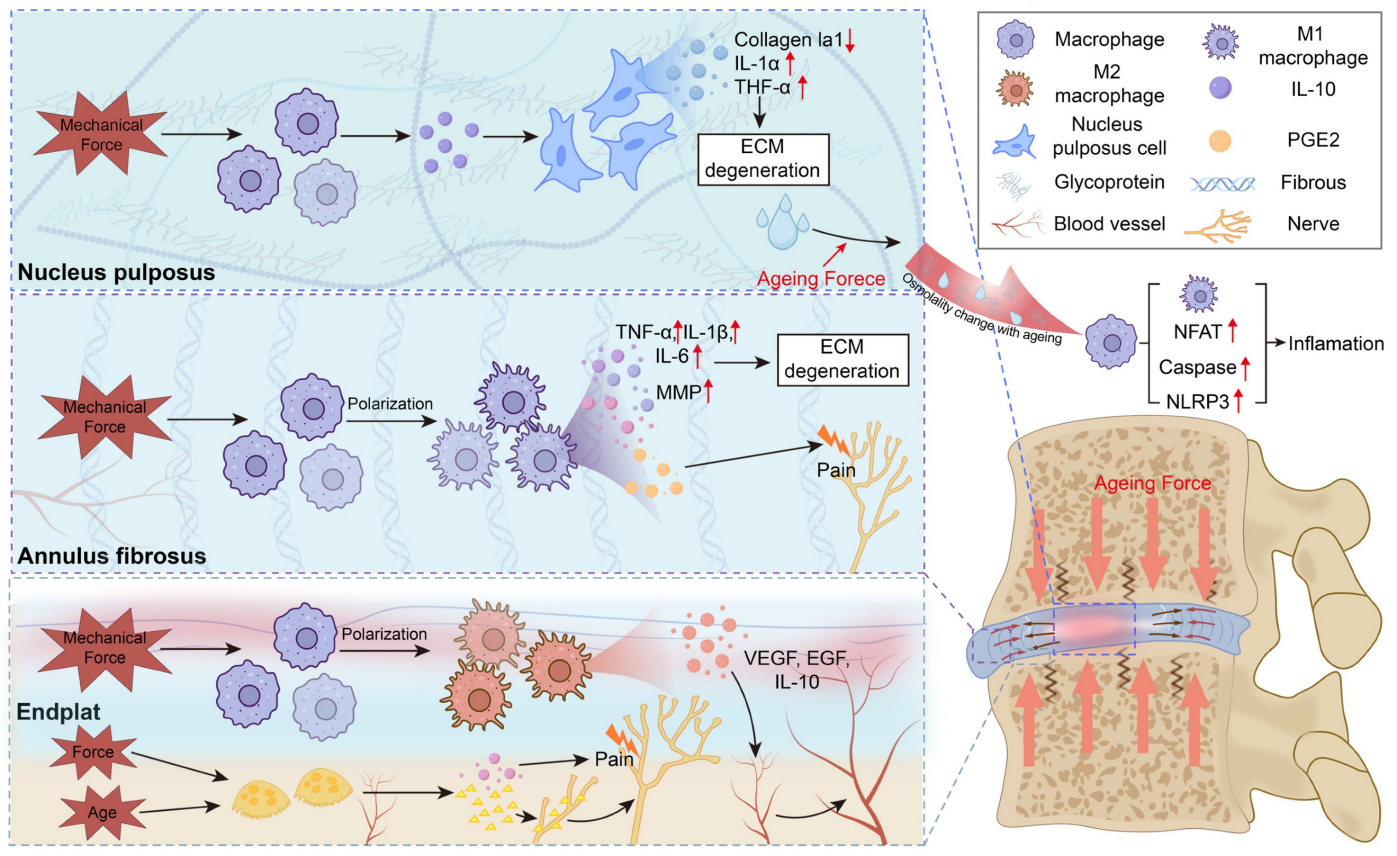
Ageing-enhanced mechanical loading drives mechano-immune dysregulation in the intervertebral disc. Ageing increases mechanical loading within the intervertebral disc, amplifying mechano-immune responses across the nucleus pulposus, annulus fibrosus, and endplate. In the nucleus pulposus and annulus fibrosus, excessive force modulates macrophage activation, promoting pro-inflammatory cytokine release and accelerating disc degeneration. In the endplate, mechanical stimulation facilitates vascular ingrowth and immune-cell infiltration, further sustaining inflammation. Moreover, ageing-related osmotic fluctuations may intensify immune-cell activation and inflammatory signaling.

IDD represents a principal contributor to chronic low back pain, affecting more than 80% of adults over the course of life and imposing substantial socioeconomic burden 221, 222. Pathogenesis involves complex interplay among mechanical overload, inflammatory activation, oxidative stress, and ECM remodeling 223, 224. Aging is accompanied by progressive degenerative alterations within the IVD, including dehydration of the NP, calcification of cartilaginous endplates, and disruption of collagen fiber organization. These changes reduce disc elasticity and alter physiological load distribution 12, 225-229. Subsequent biomechanical consequences include decreased range of motion, loss of disc height, reduced proteoglycan content and osmotic pressure, increased matrix stiffness, and heightened susceptibility of the annulus fibrosus to fissure formation. Impaired mechanical buffering capacity disrupts homeostatic mechanotransductive signaling within disc cells, reinforcing a cycle of mechanical imbalance, inflammatory activation, and matrix degradation. This self-perpetuating cascade constitutes a central mechanism underlying age-related IDD progression (Figure 5) 12, 225-229.

#### 3.3.1 Aging and Abnormal Force Disrupt the Disc Immune Barrier

Loss of immune privilege in IDD is primarily driven by the combined effects of aging and aberrant mechanical stress. Aging impairs the synthetic activity of nucleus pulposus cells (NPCs) and annulus fibrosus cells (AFCs), resulting in reduced production of proteoglycan and type II collagen, increased deposition of type I and III collagen, and upregulation of catabolic enzymes, including MMPs and ADAMTS family members 225, 230-232. These structural and metabolic alterations compromise maintenance of an immune-isolated microenvironment and lower intradiscal pressure, increasing susceptibility to stress concentration, endplate microfractures, and formation of Schmorl's nodes 12. Concurrently, aging is associated with accumulation of M2-polarized macrophages within the cartilaginous endplate. Through secretion of IL-10, these macrophages promote pathological angiogenesis and contribute to endplate stiffening 233.

Within this mechanically compromised context, abnormal mechanical loading further disrupts barrier integrity. In NPCs and annulus AFCs, excessive compressive, tensile, or shear forces induce oxidative stress, cytoskeletal remodeling, and increased expression of matrix-degrading enzyme, culminating in ECM breakdown 234-236. Chronic compression of the cartilaginous endplate promotes calcification, microdamage, and reduced permeability 237. These biomechanical insults collectively compromise matrix integrity and trigger the release of DAMPs, including high mobility group box 1 (HMGB1), ATP, and collagen fragments. DAMPs then activate Toll-like receptor- and NOD-like receptor-dependent NF-κB signaling pathways, amplifying inflammatory responses and destabilizing local immune balance 238, 239.

Collectively, aging initiates mechanical disequilibrium within the intervertebral disc, creating a permissive environment for degeneration, while progressive structural degeneration further alters mechanical loading patterns. This bidirectional reinforcement establishes a self-amplifying cycle of structural failure, immune barrier disruption, and sustained inflammation in IDD 233, 240, 241.

#### 3.3.2 Mechanical Stress Activates Disc Immune Cells

Following disruption of the cartilaginous endplate or annulus fibrosus, peripheral immune cells gain access to the intervertebral disc and are subsequently exposed to aberrant mechanical cues, including excessive compression, tensile strain, and shear stress. These altered biomechanical cues influence immune cell activation and polarization states. Mechanical overload enhances expression of matrix-degrading enzymes and, through immune-stromal crosstalk, impairs reparative capacity of NPCs and AFCs, thereby accelerating inflammatory amplification and ECM degradation.

Within the NP, mechanically induced degeneration has been associated with increased macrophage infiltration and upregulation of secreted phosphoprotein 1 (SPP1) 242. Elevated SPP1 expression inhibits the protein kinase RNA-like endoplasmic reticulum kinase (PERK)/activating transcription factor 4 (ATF4)/IL-10 signaling axis, decreasing synthesis of NPC-derived matrix components and promoting ECM breakdown. Suppression of SPP1 restores PERK/ATF4/IL-10 signaling and mitigates disc injury 242.

Regional mechanical heterogeneity further shapes immune phenotypes in degenerative discs. The high-intensity zone (HIZ) associated with annular fissures is subjected to concentrated shear and circumferential stress and is frequently characterized by granulation tissue formation, inflammatory infiltration, and neovascularization 243, 244. In contrast, Modic changes occurring at the endplate-marrow interface result from microdamage and abnormal axial loading, manifesting as marrow edema, fatty replacement, or sclerosis 245. These region-specific mechanical environments contribute to distinct immune and stromal responses within the degenerating disc.

Within the annulus fibrosus, high-intensity zone lesions are characterized by pronounced M1 macrophage polarization, creating a mechanically reinforced pro-inflammatory niche. These macrophages produce IL-6, IL-8, MMPs, and nociceptive mediators such as NGF, thereby amplifying local inflammation, accelerating ECM degradation, and enhancing pain sensitization. Mechanical stress-induced M1 programming may engage activation of the Piezo1-YAP mechanotransduction axis, a pathway implicated in pro-inflammatory macrophage activation under conditions of elevated mechanical load or matrix stiffening 94, 246, 247.

In contrast, chronic mechanical overload and endplate microfractures contribute to Modic changes, where macrophage phenotypes vary according to lesion stage. M1 macrophages predominate in acute Type I lesions, whereas M2 subsets are more prominent in chronic Type II lesions 246, 248. Early M2 responses aid debris clearance; however, prolonged M2 activity and associated SASP factors, including IL-10 and neurotrophic mediators, promote vascular and sensory nerve ingrowth, sustaining inflammation, pain, and endplate sclerosis 233, 249. Mechanical instability, whether age-related or surgically induced, further accelerates endplate degeneration by enhancing osteoclast recruitment and resorptive activity. Osteoclast-mediated micro-porosity creates structural channels for sensory fiber extension, while secretion of Netrin-1 guides nerve ingrowth and prostaglandin E_2_ (PGE_2_) sensitizes nociceptors to mechanical stimulation 250.

Although mechanobiological-immune interactions in IDD remain incompletely defined, available evidence supports a model in which aging and abnormal mechanical loading progressively transform the relatively immune-privileged disc into a mechano-inflammatory niche. This altered environment reprograms infiltrating immune cells, compromises reparative capacity of NPCs and AFCs, and facilitates pathological neurovascular ingrowth.

#### 3.3.3 Osmotic Imbalance Under Mechanical Stress May Accelerates IDD Inflammation

In the healthy intervertebral disc, osmotic pressure within the NP is maintained by proteoglycan content and associated fixed charge density, fluctuating dynamically within a physiological range of approximately 430-496 mOsm/L 251. This osmotic environment undergoes predictable diurnal variation in response to posture and mechanical loading 251. Aging is accompanied by progressive depletion of proteoglycans and reduced tissue hydration, lowering baseline osmolarity to approximately 300 mOsm/L and diminishing the intrinsic buffering and recovery capacity of the NP 119, 252, 253. Concurrent sclerosis of the cartilaginous endplate compromises water regulation and nutrient transport, further accelerating osmotic decline 254, 255. Under repetitive or sustained mechanical loading, the aging NP is therefore prone to more frequent and pronounced hypotonic fluctuations, leading to an unstable osmotic stress environment and limiting restoration of physiological balance during circadian cycles. Such osmotic dysregulation may represent an additional mechanobiological factor contributing to inflammatory activation and progression of intervertebral disc degeneration.

Osmotic fluctuations represent a critical factor in disruption IVD homeostasis. Hypoosmolar conditions activate pro-inflammatory signaling in NPCs via the TRPV4/Ca²⁺-NF-κB pathway, whereas hyperosmotic stress induces nuclear factor of activated T cells 5 (NFAT5) and NOD-like receptor family pyrin domain containing 3 (NLRP3) activation, both of which contribute to inflammatory amplification and ECM degradation 119, 252, 256. Immune cells demonstrate similarly osmotic sensitivity. Hypoosmolarity favors M1 macrophage polarization and NLRP3 inflammasome activation, whereas hypertonic conditions enhance pro-inflammatory cytokine production through NFAT5 and caspase-1 signaling pathways 257-260.

Current investigations have largely focused on static hypo- or hyperosmotic conditions. In contrast, the impact of mechanically driven, periodic osmotic fluctuations on the immune-inflammatory microenvironment of the disc remains poorly understood. In aging intervertebral discs, diminished osmotic buffering capacity of the NP may expose infiltrating immune cells to recurrent osmotic stress, thereby amplifying inflammatory polarization and tissue-destructive activity. Whether distinct immune cell subsets, including macrophages, T cells, and neutrophils, exhibit differential sensitivity to dynamic osmotic fluctuations, and how intercellular interactions evolve under such stress, represent unresolved questions that warrant systematic investigation.

### 3.4 Enthesis Pathology

Enthesis-related injuries, such as rotator cuff tears and Achilles tendinopathy, represent some of the most prevalent musculoskeletal disorders, with incidence increasing markedly with advancing age. Epidemiological data indicate that structural or inflammatory abnormalities at the enthesis are present in more than 70% of middle-aged adults, and degeneration of enthesis fibrocartilage is strongly associated with pain, muscle weakness, and reduced mobility 261, 262.

The enthesis is characterized by a graded transitional architecture that enables efficient force transfer between tendon and bone. Repetitive mechanical loading or acute trauma disrupts this gradient structure, resulting in microtears, fibrocartilage disorganization, and compromised healing capacity 262, 263. Aging further exacerbates structural vulnerability. The fibrocartilaginous transition zone becomes thinner and architecturally disordered, cellularity declines, and mineralization expands, collectively reducing elasticity and mechanical resilience 264, 265. Increased collagen cross-linking, diminished viscoelastic properties, and dysfunction of resident stem or progenitor cells further lower the mechanical tolerance of the tendon-bone interface 264, 265. Under these conditions, even physiological loading may induce microdamage and release DAMPs, such as high mobility group box 1 (HMGB1) and ATP. These signals activate macrophage-mediated inflammatory responses and promote M1 polarization, thereby contributing to chronic enthesis pathology 266-268.

The magnitude, pattern, and timing of mechanical stimulation critically determine the nature of the immune response at the enthesis. Controlled and appropriately timed loading favors resolution of inflammation by promoting macrophage transition toward an M2 phenotype and supporting coordinated ECM remodeling. In contrast, premature, excessive, or irregular mechanical stress prolongs the M1-dominant inflammatory phase and predisposes to fibrotic repair rather than functional regeneration 269, 270. Aging attenuates this adaptive flexibility. Macrophages in aged tissues exhibit reduced capacity for M1-to-M2 phenotypic transition, resulting in sustained inflammation, progressive ECM degradation, and fibrotic remodeling of the tendon-bone interface 4, 271.

Such age-associated alterations define an “aging-mechanical-immune” axis, in which impaired mechanotransduction and unresolved inflammatory signaling reinforce perpetuate tendon-bone degeneration. Therapeutic strategies directed toward this axis—including optimized mechanical conditioning protocols, targeted modulation of macrophage polarization, and application of bioengineered scaffolds designed to restore physiological mechanical cues—may offer potential to re-establish enthesis homeostasis and improve tendon-to-bone healing in aging individuals.

### 3.5 Sarcopenia

Sarcopenia, characterized by progressive loss of skeletal muscle mass and functional capacity, represents a major contributor to frailty, falls, and disability in older adults. Prevalence ranges from 5-13% in individuals aged 60-70 years and approaches 50% in those older than 80 years 272, 273. Although multifactorial in origin, immune dysregulation has emerged as a key component of pathogenesis. Aging reshapes the muscle immune microenvironment, defined by metabolic reprogramming of macrophages, impaired transition from pro- inflammatory to reparative phenotypes, and reduced Treg function 274.

Age-associated alterations in mechanosensitivity and polarization dynamics further increase susceptibility of skeletal muscle to degeneration during cycles of mechanical unloading and reloading, such as disuse, immobilization, or microgravity exposure. In young muscle, reloading after atrophy induces transient M1 macrophage activation to facilitate clearance of necrotic debris, followed by polarization toward an M2 phenotype that secretes insulin-like growth factor 1 (IGF-1) and IL-10, thereby promoting regeneration. In aged muscle, this M1-to-M2 transition is attenuated, delaying recovery and impairing regenerative efficiency 274-278. Immobilization reduces the abundance of inducible nitric oxide synthase (iNOS)⁺ macrophages, suppresses iNOS expression, and disrupts satellite cell activation, collectively compromising myofiber regeneration. Prolonged mechanical unloading also sustains Ly-6C⁺ inflammatory macrophages and inhibits transition to anti-inflammatory phenotypes, leading to sustained TNF-α and IL-1β production that exacerbates inflammation and accelerate muscle atrophy 279. Restoration of the pro-inflammatory-to-reparative shift, achieved through macrophage transplantation or targeted metabolic and immunomodulatory interventions, including transient hypoxia or metabolic reprogramming, has been shown to enhance muscle regeneration and functional recovery in aged, disused models 280, 281.

## 4. Strategies for Modulating Musculoskeletal Mechano-Immunity

### 4.1 Mechanical and Material-Based Interventions

Mechano-immunity plays a dual role in the musculoskeletal system, sustaining tissue homeostasis under physiological conditions while contributing to pathological progression in degenerative disorders. Therapeutic modulation of this axis has therefore emerged as a promising strategy. Application of controlled external mechanical stimuli may reshape the immune microenvironment during early disease stages or counteract aberrant mechanical signaling in advanced degeneration. Current approaches can be broadly categorized into three principal strategies: exercise-based interventions, exogenous physical stimulation modalities, and development of advanced biomaterials designed to restore or optimize mechanical-immune coupling. Among these, structured exercise regimens remain the most extensively studied and mechanistically characterized modality (Table 3).

#### 4.1.1 Exercise Therapy

Exercise confers protective effects on musculoskeletal tissues by optimizing biomechanical loading patterns and modulating the local immune microenvironment. Clinical evidence demonstrates that a 12-week Tai Chi program performed three times weekly improves pain and functional outcomes, accompanied by enhanced neuromuscular control, improved load distribution, and regulation of inflammatory and immune pathways 282. Comparable benefits have been observed with structured yoga and resistance-based strengthening programs 283. Whole-body vibration training (WBV; 35-40 Hz, 4 mm amplitude, 3 sessions per week) combined with squat exercises further augments T cell-mediated immune responses and delays progression of knee OA in older adults 284.

Preclinical studies provide mechanistic support for these findings. Moderate-intensity treadmill running (10-20 m/min, 30 minutes per day) increases intra-articular lipoxin A4 levels, promotes macrophage polarization toward an M2 phenotype, and reduces synovitis in mid-stage OA models 74, 285. Low-intensity running similarly attenuates disease progression in aged mice by suppressing synovial inflammation and enhancing M2 polarization. 286. Prolonged moderate running (18 m/min for 90 minutes) expands intramuscular Treg populations, restrains IFN-γ production, and preserves mitochondrial integrity 26. Endurance training protocols (45 minutes per session, 4 days per week for 4 weeks) increase M2 and M2c macrophage subsets, stimulate myofiber hypertrophy, and enhance satellite cell accumulation via upregulation of hepatocyte growth factor (HGF)/insulin-like growth factor 1 (IGF1), alongside suppression of CCAAT/enhancer-binding protein β (C/EBPβ) and muscle RING finger protein through the TWEAK-FN14 signaling axis 79.

In tendon tissue, exercise-induced mechanical loading activates IL-4/JAK/STAT6 signaling, driving macrophage polarization toward an M2 phenotype and facilitating fibrocartilage regeneration. Eccentric loading protocols (10 m/min, 20 minutes per day, 2-8 weeks) reduce M1 macrophage prevalence and accelerate tendon-bone healing in rotator cuff injury models 86, 270.

Exercise also exerts systemic osteoimmune effects via the muscle-bone axis. A regimen of treadmill running (12 m/min, 1 hour per day, 8 weeks) enhances skeletal-immune regulation. Contracting muscle releases myogenic metabolites, such as L-β-aminoisobutyric acid (L-BAIBA), which suppress osteoclastogenesis and prevent bone loss in ovariectomized models 287. Within the bone marrow niche, mechanical stimulation increases macrophage-derived RCN2, which interacts with neuregulin-2 and integrin β1 on adipocytes to activate the cAMP-PKA pathway, thereby promoting lipolysis and generating metabolic substrates that support osteogenesis 61. Sustained exercise further preserves LEPR^+^ osteolectin^+^ matrix cells, maintaining pools of osteogenic and lymphoid progenitors and reinforcing skeletal-immune homeostasis 27.

Adults aged ≥75 years often experience declines in muscle strength, balance, and overall functional capacity, limiting tolerance for high-intensity exercise. Clinical evidence shows that low- to moderate-intensity activities—such as Tai Chi, walking, chair-based exercise, or structured multicomponent programs—performed for approximately 150 minutes per week (e.g., 30 minutes per session, 5 sessions per week) at moderate exertion level ( approximately 55-75% of maximal heart rate) safely improve muscular strength, balance, flexibility, and functional capacity in this age group 282, 288, 289. Mechanistic studies further demonstrate that graded, low- to moderate-load mechanical stimuli effectively engage mechano-immune pathways, including promotion of anti-inflammatory macrophage polarization, expansion of T cell populations, and enhancement of tissue repair processes. These converging findings support the concept that appropriately prescribed, low- to moderate-intensity exercise in advanced age may exert therapeutic benefit through activation of mechano-immune mechanisms, thereby linking underlying physiological mechanisms with feasible clinical applications.

#### 4.1.2 Exogenous Physical Stimulation

In addition to conventional exercise, externally applied mechanical stimuli are increasingly investigated as therapeutic “mechanical prescriptions” capable of reprogramming immune responses. Modalities such as low-intensity pulsed ultrasound (LIPUS), mechanical vibration, and controlled stretching activate cellular mechanosensors, including Piezo1 and TRPV4, with activation efficiency dependent on stimulus frequency, intensity, duty cycle, and duration. In vitro experiments indicate that LIPUS delivered at 38 kHz, 250 mW/cm², 20% duty cycle for 90 min optimally activates Piezo1 in macrophages and promotes anti-inflammatory polarization. In contrast, in vivo applications in preclinical and clinical settings commonly employ higher frequencies and lower intensities, such as 1 MHz, 30 mW/cm², 20% duty cycle for 10 minutes per day, which are sufficient to induce Piezo1-mediated Ca²⁺ influx in immune and osteolineage cells 290-292. Low-amplitude, high-frequency vibration (20-40 Hz, 0.3-1.0 g, 10-20 minutes per session) has similarly been shown to gate Piezo1 and TRPV4 channels 293.

Distraction osteogenesis (DO) provides a clinically established example of mechanically guided tissue regeneration. Standard protocols typically involve a latency period of 5-7 days, a distraction rate of approximately 1 mm per day applied in multiple incremental steps, and a consolidation phase of at least 6-8 weeks, collectively promoting stable bone formation and neovascularization. However, direct characterization of mechanosensitive receptor activation, including Piezo1 and TRP channels, under these clinical parameters remains limited. Most mechanistic insights are derived from in vitro or animal models, whereas systematic quantification linking distraction rate, rhythm, and strain magnitudes to mechanoreceptor activation remains lacking 58, 294.

Therapeutic modulation of mechano-immune pathways through externally applied mechanical stimuli has been validated in multiple experimental models. Manual massage (10-30 minutes per day at 1 Hz) promotes macrophage polarization toward an M2 phenotype while suppressing M1 dominance in young murine models, accelerates muscle recovery, and enhances neutrophil clearance to preserve tissue homeostasis 78, 295, 296.

LIPUS similarly exploits mechanosensitive signaling pathways to regulate immune responses. Application of LIPUS facilitates tendon-bone interface repair by promoting anti-inflammatory M2 macrophages polarization 297. In the DMM OA model, LIPUS enhances macrophage autophagic activity, facilitates degradation of pyruvate kinase M (PKM) and sequestosome 1 (SQSTM1), reduces mature IL-1β levels, and improves gait parameters while attenuating synovial inflammation 298. Suppression of osteoclast activity and reduced Netrin-1 secretion further contribute to analgesic effects 299.

Mechanical vibration represents an additional modality with mechanotype-specific effects. Low-amplitude high-frequency vibration (LMHFV) promotes bone healing in ovariectomized osteoporotic fracture models by modulating macrophage polarization 300. Nanovibrational stimulation has been demonstrated to inhibit osteoclastogenesis and enhance osteogenic differentiation, supporting bone regeneration under mechanically optimized conditions 301.

DO represents a classical endogenous bone engineering approach in which controlled mechanical distraction, applied at defined rates and frequencies, induces tissue regeneration 302. Mechanical stretching during DO activates FAK/MAPK, PI3K/AKT, and Wingless-related integration site (Wnt) signaling pathways, shifts macrophage polarization toward anti-inflammatory phenotypes, and upregulates TGF-β, BMP, and IL-6 expression, thereby promoting angiogenesis and osteogenesis 303. Mechanical stretching has also shown anti-inflammatory effects in intervertebral disc degeneration models, potentially through analogous mechanically regulated immune mechanisms 304, 305.

Clinical cohort studies indicate that early application of LIPUS is associated with favorable fracture healing outcomes in elderly patients, with advanced age not substantially diminishing therapeutic efficacy, supporting its relevance in geriatric bone repair 306. WBV (20-40 Hz, 1-4 mm amplitude, 0.3-1.0 g, 10-20 minutes per session) improves muscle strength and balance in older adults. Notably, increases in bone mineral density are more pronounced in individuals with osteopenia or osteoporosis, whereas effects in healthy young adults are generally minimal, suggesting preferentially benefit in mechanically or metabolically compromised bone 307-309. Clinical observations further indicate that bone regeneration can be achieved across age groups with DO, although prolonged consolidation periods are often required in elderly patients 310. Collectively, WBV and DO remain clinically applicable in older populations under standard parameters; however, regenerative efficiency may be slightly lower compared with younger individuals, consistent with age-associated decline in mechanosensitivity. Integration of multiple physical modalities, including exercise combined with DO or WBV, may enhance regenerative responses and optimize outcomes in aging musculoskeletal tissues.

#### 4.1.3 Mechanical Stimulation of Bionic Materials and Scaffolds

Micromotion fixation systems are designed to provide controlled sub-millimeter axial displacement at fracture sites, thereby maintaining local strain within a biologically permissive range. In contrast to rigid fixation, such systems preserve moderate interfragmentary micromotion, supporting periosteal mechanosensing and facilitating early immune regulation during bone repair 311-313. Experimental evidence shows that intermediate compressive strain of approximately 5%, comparable to micromotion-induced strain, promotes a regenerative macrophage phenotype, suppresses M1-dominant inflammation, and enhances pro-angiogenic signaling. In contrast, excessive strain approaching 35% triggers maladaptive inflammatory responses and impairs mineralization processes 314. Maintenance of strain within this optimal mechanobiological window likely enables coordinated macrophage-mediated angiogenic and osteogenic coupling, thereby improving fracture healing outcomes 314.

Bionic materials and scaffold-derived mechanical cues have emerged as important modulators of immune responses in musculoskeletal repair. Implants engineering with moderate stiffness (approximately 80 kPa) inhibit Piezo1 activity and downregulate the IL-17/TNF signaling axis, thereby reducing inflammation associated with senescent macrophage while enhancing osteogenesis and implant integration 315. These findings suggest that rationally designed mechanical properties can alleviate local inflammatory activation and potentially mitigate features of immunosenescence. Low-modulus Ti2448 alloy similarly modulates macrophage Piezo1 via stiffness-dependent cues, activating the Piezo1-YAP pathway to promote M2 polarization, angiogenesis, and osteogenic differentiation 316. Surface nano-sheet arrays further stimulate integrin β2/FAK-PI3K-AKT-mTOR signaling, driving macrophage polarization toward M2 phenotype and supporting bone regeneration 317. Left-handed nanohydroxyapatite activates the integrin β1-FAK-TRPV4 axis to reprogram macrophage responses and enhance osteogenic activity 314. In tendon repair models, ordered nanofiber topographies modulate macrophage-driven inflammatory responses, and when combined with tensile loading, further promote M2 polarization to improve structural and functional healing outcomes 318, 319.

Beyond intrinsic physicochemical characteristics, emerging bionic and piezoelectric materials are capable of converting mechanical stimuli into bioactive immunoregulatory signals. Piezoelectric hydrogels combined with structured mechanical training have been demonstrated to accelerate rotator cuff repair and improve mechanical strength of the repaired interface 320. Piezoelectric bioadhesives subjected to mechanical stress activate TRPV1-Ca²^+^-cAMP signaling, thereby promoting macrophage polarization toward an M2 phenotype, enhancing angiogenesis, and facilitating fibrocartilage formation 321. In bone regeneration models, magnetically responsive hydrogels exposed to static magnetic fields stimulate M2 macrophage differentiation and enhance osteogenesis through activation of the podosome/Rho/ROCK pathway 322. Scaffolds integrating LIPUS with piezoelectric components further augment Piezo1-mediated mechanotransduction, promote M2 polarization, and support bone regeneration 290, 323. Advanced bioreactor systems capable of delivering precisely controlled mechanical stimuli to macrophages have recently been developed to generate EVs enriched with defined bioactive cargo, including miR-210-3p. These mechanically conditioned vesicles markedly accelerate bone defect healing in preclinical models, offering a promising cell-free therapeutic approach for musculoskeletal regeneration 324.

Despite accumulating evidence that mechanical cues regulate immune programs during tissue repair, the mechanistic framework and translational potential of mechano-immunomodulation in chronic musculoskeletal disorders, such as osteoarthritis and sarcopenia, remain incompletely defined. Advancement in this field will likely require development of tissue-specific “mechanical prescriptions” that target distinct mechano-immune axes accordingly to pathological context. For synovial joints, multidirectional, low-impact movement strategies such as Tai Chi and yoga may optimize load redistribution and alleviate synovial inflammation 282, 283. In skeletal muscle, combined endurance and resistance training protocols may restore myokine signaling and facilitate T cell- and macrophage-mediated regenerative processes 325, 326. Comparable precision-based mechanical interventions tailored to bone, tendon, and intervertebral disc tissues may similarly re-establish local immune equilibrium and improve functional recovery. Refinement of such targeted strategies may provide a framework for integrating mechanobiology with immune modulation in the management of age-related musculoskeletal disease.

### 4.2 Precision Targeting of Mechano-Immune Signaling Networks

Although mechanical loading, structured exercise, and biomaterial-derived stiffness provide effective means for modulating mechano-immunity at the tissue level, a complementary and potentially more precise strategy involves direct targeting of mechanoreceptors and their downstream epigenetic regulatory elements.

#### 4.2.1 Targeting Mechanosensitive Ion Channels in Immune Cells

Mechanosensitive ion channels, particularly Piezo1, represent promising molecular targets for modulation of immune cell mechanotransduction. Pharmacological inhibition of Piezo1 with the peptide GsMTx4 has been reported to attenuate disease progression in ankylosing spondylitis through regulation of macrophage autophagy 327. Natural compounds and traditional Chinese medicine-derived formulations have also demonstrated modulatory effects on Piezo1 signaling. Guizhitongluo Tablet has been observed to reduce macrophage Piezo1 expression and Ca²⁺ influx, thereby suppressing NLRP3 inflammasome activation and pyroptosis, ultimately delaying atherosclerosis progression in murine models 328. The alkaloid jatrorrhizine similarly inhibits Piezo1 channel activation and mitigates vascular inflammation 329. These findings suggest that modulation of Piezo1-dependent mechanosensing pathways may provide a strategy for regulating mechano-immune responses in chronic inflammatory states. Bioactive compounds derived from herbal sources may offer comparatively favorable safety profiles while influencing mechanotransductive signaling. Nevertheless, targeted manipulation of immune cell Piezo1 in degenerative musculoskeletal disorders remains insufficiently investigated, and translation of such approaches into therapeutic application is currently at an early experimental stage.

#### 4.2.2 Targeting Mechanosensitive Epigenetic Modifications

Aberrantly regulated enhancers have been identified in degenerative musculoskeletal disorders including osteoporosis, IDD, and osteoarthritis, implicating epigenetic dysregulation in disease pathogenesis 330-332. Recent genomic analyses have delineated mechanosensitive enhancers responsive to ECM stiffness, including loci associated with SKP2, connective tissue growth factor (CTGF), and myosin heavy chain 9 (MYH9). These regulatory elements modulate transcriptional programs governing apoptosis, proliferation, migration, and cytoskeletal contractility, thereby establishing a direct molecular interface between mechanical cues and gene expression^333^.

Despite these advances, therapeutic exploitation of mechanosensitive enhancers within immune cells in musculoskeletal tissues remain largely unexplored. Epigenetic modulation of enhancer activity has emerged as a promising strategy. Pharmacological agents targeting bromodomain and extraterminal (BET) proteins or DNA methyltransferases (DNMTs) have demonstrated the capacity to regulate disease-associated enhancer landscapes and have shown efficacy in oncologic and cardiovascular contexts. 334. Translation of such approaches to mechano-immune regulation in musculoskeletal disorders warrants systematic investigation.

Future directions may include precise gene or epigenome editing to directly regulate mechanoresponsive enhancer elements, as well as biomaterial-based approaches, such as stiffness-tunable hydrogels. to alter the mechanical microenvironment and indirectly reshape enhancer activity. Integration of these approaches may provide a framework for precision-based intervention in degenerative musculoskeletal diseases.

#### 4.2.3 Targeting the Bone Mechanical-Neural-Immune Axis

Intraosseous interoception has recently been recognized as a regulatory mechanism mediated by Aδ and C sensory fibers that detect mechanical forces and metabolic stress within bone tissue, thereby contributing to skeletal homeostasis 335. Under pathological conditions, such as aging and diabetes, intraosseous sensory function declines, leading to impaired mechanosensory signaling and associated immune dysregulation 336. Restoration of disrupted intraosseous interoception through advanced biomaterials strategies may therefore improve both bone metabolic activity and the local immune microenvironment.

Material-based strategies aimed at re-establishing intraosseous interoceptive function include natural or synthetic carriers delivering neurotrophic factors or neuropeptides, inorganic or metal-based biomaterials releasing bioactive ions, conductive composite materials, and cell-free biologic constructs. These platforms promote reconstruction of the intraosseous sensory network by activating nerve-bone signaling pathways, promoting nerve reinnervation, and facilitating transmission of mechanical signals. Detailed overviews of these strategies have been provided in recent reviews 337-339.

Collectively, precision targeting of mechano-immune networks across multiple regulatory layers—including mechanosensitive ion channels, epigenetic enhancer elements, and the bone mechanical-neural-immune axis—represents a multifaceted framework for restoring tissue homeostasis. Although most approaches remain at the preclinical stage, such strategies outline a conceptual basis for future translational therapies in degenerative musculoskeletal diseases.

## 5. Conclusion

Aging exerts profound effects on the musculoskeletal system through tightly interconnected mechanical and immune pathways. Progressive reductions in tissue elasticity, ECM integrity, and mechanosensory capacity of immune and stromal cells disrupt physiological mechano-immune signaling, fostering chronic inflammation and structural degeneration in bone, joints, skeletal muscle, tendon, and intervertebral disc tissues. These alterations establish self-reinforcing feedback loops in which mechanical imbalance intensifies immune dysfunction, while immunosenescence further impairs mechanotransductive responsiveness.

Recognition of these age-associated mechano-immune interactions provides a unifying framework for understanding the progression of osteoporosis, OA, IDD, and sarcopenia. This perspective also identifies potential therapeutic avenues. Restoration or recalibration of mechano-immune coupling through controlled mechanical stimulation, biomaterial-guided mechanoregulation, and targeted modulation of cellular mechanotransductive signaling networks may attenuate degenerative cascades, promote tissue repair, and improve functional outcomes in older populations. Continued integration of mechanobiology, immunology, and tissue engineering will be pivotal for translating principles of mechano-immunity into precision-based interventions for age-related musculoskeletal disorders.

## Figures and Tables

**Table 1:** Summary of mechanosensing and mechanotransduction structures in immune cells.

Category	Representative Molecules	Sensed Mechanical Stimuli	Signal Transduction Mechanism	Downstream Pathways	Reference
Adhesion receptors	Integrins (β1, β2, αvβ3, LFA-1)	ECM stiffness, tensile strain, shear stress	Force-induced clustering activates FAK, Src, and PLC, initiating phosphorylation cascades	MAPK, NF-κB, and YAP/TAZ activation; cytoskeletal remodeling	35, 40, 41
Immune receptors	TCR, BCR	Cell-cell traction, antigen stiffness	Receptor-ligand mechanical deformation triggers ITAM phosphorylation and Ca²⁺ influx	NFAT, NF-κB, ERK activation	33, 34
Mechanosensitive ion channels	Piezo1, TRPV4, K2P (TREK-1, TRAAK)	Membrane stretch, pressure, compression, osmotic and shear stress	Ion influx (Ca²⁺, Na⁺, K⁺) activates CaMKII, calcineurin, and PKC signaling	NFAT, NF-κB, YAP/TAZ; metabolic reprogramming	31, 32, 39, 42
Cytoskeletal network	Actin filaments, microtubules, intermediate filaments	Tensile/compressive stress, substrate rigidity	Transmission of mechanical tension via RhoA-ROCK and FAK pathways	Nuclear deformation; activation of YAP/TAZ, NF-κB	46, 47
Cytoskeleton-nucleus coupling	LINC complex (nesprin-SUN), lamins A/C	Cytoskeletal tension, substrate stiffness	Force transmission to nucleus; modulation of chromatin accessibility and nuclear stiffness	Transcriptional regulation; mechanosensitive gene expression	43-45

ECM, extracellular matrix; FAK, focal adhesion kinase; PLC, phospholipase C; MAPK, mitogen-activated protein kinase; NF-κB, nuclear factor κB; YAP, Yes-associated protein; TAZ, transcriptional co-activator with PDZ-binding motif; TCR, T-cell receptor; BCR, B-cell receptor; ITAM, immunoreceptor tyrosine-based activation motif; CaMKII, calcium/calmodulin-dependent protein kinase II; PKC, protein kinase C; NFAT, nuclear factor of activated T cells; TRPV4, transient receptor potential vanilloid 4; TRPV2, transient receptor potential vanilloid 2; ASICs, acid-sensing ion channels; K2P, two-pore potassium channels; TREK-1, TWIK-related K⁺ channel 1; TRAAK, TWIK-related arachidonic acid-activated K⁺ channel; ROCK, Rho-associated kinase; LINC, linker of nucleoskeleton and cytoskeleton; SUN, Sad1/UNC-84 domain protein.

**Table 2:** Summary of mechano-immune regulation mechanisms maintaining musculoskeletal homeostasis.

Tissue	Immune Cell Type	Mechanical Stimulus	Mechanistic Pathway	Biological Effect	Ref.
Bone (Periosteum)	CD68⁺F4/80⁻ myeloid cells / macrophages (mouse)	Cyclic compression (0.1 Hz, 10 kPa, 1 h/day, 3 days)	Piezo1 activation induces differentiation of CD68⁺F4/80⁻ precursors into CD68⁺F4/80⁺ macrophages, leading to TGF-β1 secretion	Recruits bone progenitors and promotes cortical bone formation	52
	BMDMs (mouse)	Shear stress (1 Pa, 2 h, orbital shaker)	Mechanical induction of periostin expression promotes macrophage M2 polarization and TGF-β release	Enhances angiogenesis and BMSC-mediated osteogenesis during bone repair	53-55
Bone (Marrow)	BMDMs (rat)	Treadmill running (10 m/min, 30 min/day, 21 days)	Macrophage-derived reticulocalbin-2 (RCN2) activates Neuropilin-2 / Integrin β1 / cAMP-PKA signaling	Induces lipolysis in bone-marrow adipocytes and enhances osteogenesis and hematopoiesis	61
	BMDMs (mouse)	Uniaxial strain (10%, 0.5 Hz, 0-10 h)	Mechanical stimulation triggers release of UCHL3-enriched exosomes, activating Smad1-RUNX signaling in BMSCs	Promotes mesenchymal stem cell osteogenesis	63
	BMDMs (mouse)	Tibial loading (2 Hz, 1-6 N, 120 cycles)	Piezo1 activation induces VEGFA and BMP-2 secretion	Enhances vessel-bone coupling and bone regeneration	59
	RAW264.7 cells	Cyclic stretch (10%, 0.5 Hz, 0-6 h)	Piezo1 activation suppresses p53 signaling, promoting M2 polarization and TGF-β1 secretion	Enhances osteogenic differentiation of BMSCs	58
	RAW264.7 cells	Cyclic stretch (10%, 0.5 Hz, 0-7 h)	Mechanical stimulation induces mitochondrial fission and CD200R-CD200 vesicular signaling	Upregulates Runx2, Osx, and ALP in MSCs, promoting osteogenesis	64
	γδ T cell-associated macrophages (mouse)	Orthodontic tooth movement	Mechanical stress induces macrophage IL-17A secretion, stimulating fibroblast RANKL expression and monocyte/neutrophil recruitment	Regulates bone remodeling	66
	dHL-60 cells (human)	Cyclic stretch (0.5 Hz, 8-12% strain, 6 h)	PI3K-AKT activation in neutrophil-lineage cells induces Oncostatin M secretion	Promotes bone regeneration	67
Joint	Synovial macrophages (mouse)	Dynamic sinusoidal loading (1 N, 5 Hz, 6 min/day, 2 weeks)	Mechanical load suppresses PI3K/AKT and NF-κB pathways, promoting M2 polarization	Increases IL-10 and TGF-β, reduces MMP-13, and protects cartilage matrix	73
	Synovial macrophages (human)	Cyclic strain (10%, 0.5 Hz, 24 h) with 1 nM LXA₄	LXA4 promotes M2 polarization, increases IL-10 and TGF-β1, and suppresses TNF-α and IL-1β via NF-κB/NLRP3 inhibition	Attenuates inflammation and maintains synovial homeostasis	74
	Osteoclasts (mouse)	Dynamic load (1 N, 5 Hz, 5 min/day, 2 weeks)	Mechanical stimulation upregulates Wnt3a and downregulates NFATc1, RANKL, TNF-α, and cathepsin K	Suppresses osteoclastogenesis and preserves subchondral bone integrity	75
Tendon	Macrophages (mouse)	Downhill treadmill running (-15°, 10 m/min, 20 min/session, 5 days/week, 5 weeks)	Eccentric loading promotes macrophage M2 polarization and TGF-β secretion	Enhances fibroblast proliferation and tendon-bone interface healing	86
Muscle	Regulatory T cells (mouse)	Voluntary wheel running	IL-6Rα signaling induces a highly functional muscle-resident Treg phenotype with elevated Areg, EGFR, and ST2 expression	Suppresses inflammation and promotes muscle regeneration	81
	Muscle spindle macrophages (mouse)	Muscle contraction	Uptake of glutamine and conversion to glutamate modulate sensory nerve activity	Maintains muscle stretch reflex and neuromuscular balance	82
	Neutrophils (mouse)	Cyclic mechanical loading (1 Hz, 0.15-0.6 N, 10 min/day, 14 days)	Mechanical compression induces CXCL2, MMP-9, and CCL3 secretion, reducing neutrophil recruitment	Resolves inflammation and promotes muscle recovery	78
	Skeletal-muscle macrophages (human)	Endurance training	Exercise enhances macrophage M2 polarization, increases IL-4, and suppresses IL-6 expression	Promotes muscle hypertrophy and satellite-cell proliferation	79, 80
Intervertebral Disc	-	-	-	-	-

AKT, protein kinase B; ALP, alkaline phosphatase; BMDMs, bone marrow-derived macrophages; BMP-2, bone morphogenetic protein-2; CCL3, C-C motif chemokine ligand 3; CD68, cluster of differentiation 68; CXCL2, C-X-C motif chemokine ligand 2; cAMP, cyclic adenosine monophosphate; EGFR, epidermal growth factor receptor; F4/80, EGF-like module-containing mucin-like hormone receptor-like 1; IL, interleukin; IL-10, interleukin-10; IL-17A, interleukin-17A; LXA₄, lipoxin A₄; MMP-9, matrix metalloproteinase-9; NLRP3, NOD-like receptor family pyrin domain-containing 3; NFATc1, nuclear factor of activated T cells 1; NF-κB, nuclear factor κB; PI3K, phosphoinositide 3-kinase; PKA, protein kinase A; RANKL, receptor activator of NF-κB ligand; RCN2, reticulocalbin-2; RUNX, Runt-related transcription factor; Smad, mothers against decapentaplegic homolog; ST2, suppression of tumorigenicity 2; TGF-β, transforming growth factor beta; TNF-α, tumor necrosis factor alpha; UCHL3, ubiquitin carboxyl-terminal hydrolase L3; VEGFA, vascular endothelial growth factor A; Wnt3a, wingless-type integration site 3A.

**Table 3:** Summary of mechanobiology-based immunomodulatory interventions for treating degenerative musculoskeletal diseases.

Category	Mechanical Intervention Type	Animal / Cell Model	Cellular Target	Mechanistic Pathways	Biological Effects	Ref.
Exercise	Moderate treadmill running (10-20 m/min, 30 min/day, 3×/week, for 4 weeks)	MIA-induced OA rats	Synovial macrophages	Promotes M2 polarization via upregulation of IL-10 and TGF-β, and inhibition of NF-κB and NLRP3 activation	Reduced joint inflammation and chondrocyte pyroptosis, preserved cartilage matrix	74, 285
	Low-intensity treadmill running (10 m/min, 15 min/day for 3 days)	SAMP8 spontaneous OA mice	Synovial macrophages	Decreases M1 macrophage infiltration and downregulates MCP-1 and TNF-α expression	Reduced synovitis and cartilage degradation	286
	Whole-body vibration+squat (35-40 Hz, 4 mm, 3×/week)	Elderly OA patients	CD4⁺ T cells	Modulates T-cell immune responsiveness	Potential slowing of osteoarthritis progression	284
	Endurance running (18 m/min, 90 min)	Exercise-induced muscle inflammation	Regulatory T cells (Tregs)	Expands muscle Tregs, suppresses IFN-γ production, and maintains mitochondrial electron transport chain function	Preserved mitochondrial metabolism and exercise capacity	26
	Endurance cycling (45 min, 4 days/week, 4 weeks)	Human participants	Macrophages	Increases IL-10 and decreases TNF-α expression; activates M2 polarization and interaction with muscle satellite cells	Enhanced M2 macrophage population, muscle hypertrophy, and repair	79
	Eccentric mechanical stimulation (downhill treadmill)	Mouse rotator cuff injury model	Macrophages	Increases M2 macrophage expression and decreases M1 macrophage expression	Enhanced fibrocartilage formation and proteoglycan content	86
	Moderate treadmill exercise (10 m/min, 20 min/day,2-8 weeks)	Mouse rotator cuff injury model	Macrophages	Activates the IL-4/JAK/STAT6 pathway, promoting M2 polarization (elevated CD206, Arg-1, IL-10)	Enhanced fibrocartilage formation and tendon-bone integration	270
	Treadmill running (12 m/min, 1 h/day, 8 weeks)	OVX osteoporotic mice	Osteoclast precursors	Muscle-derived L-BAIBA through SLC6A6 suppresses PI3K/AKT/NF-κB signaling and activates NRF2	Decreased osteoclastogenesis and bone loss	287
	Voluntary running	Mouse model	LEPR⁺/Osteolectin⁺ stromal cells	Muscle-derived L-BAIBA suppresses PI3K/AKT/NF-κB signaling and activates NRF2	Supports osteogenesis and lymphopoiesis	27
	Treadmill running (10 m/min, 30 min/day, 3 weeks)	Mouse model	Macrophages	RCN2 activates Neuropilin-2/Integrin β1-cAMP-PKA signaling	Promotes lipolysis, osteogenesis, and hematopoiesis	61
External mechanical stimulation	Robot-actuated cyclic loading (0.15-0.3 N, 1 Hz, 10 min/day) with anti-inflammatory therapy	Aged mouse tibialis anterior muscle injury model	Macrophages, muscle stem cells	Combined loading and therapy suppress NF-κB activation and restore YAP/MRTF-A mechanotransduction	Enhanced muscle regeneration, vascularization, and contractile strength	295
	Cyclic compressive loading (robotic actuator)	Mouse tibialis anterior muscle injury	Neutrophils	Mechanical loading accelerates neutrophil clearance and downregulates MMP-9 and CCL3	Improved muscle healing and biomechanical strength	296
	LIPUS (1.5 MHz frequency, 1:4 duty cycle, and 30 mW/cm²)	Rat rotator cuff repair model	Macrophages	Promotes early macrophage accumulation followed by M2 transition	Inhibits osteoclastogenesis and enhances osteogenesis	297
	LIPUS (1.5 MHz frequency, 1:4 duty cycle, and 30 mW/cm²)	DMM-induced OA model	Macrophages	SQSTM1-dependent autophagic degradation of PKM2 reduces IL-1β maturation	Anti-inflammatory effect and improved joint function	298
	LIPUS (1.5 MHz frequency, 1:4 duty cycle, and 30 mW/cm²)	Rat ACLT post-traumatic OA model	Osteoclasts	Suppresses netrin-1 expression and osteoclast differentiation	Mitigates cartilage degradation and pain, preserves bone structure	299
	Low-magnitude high-frequency vibration (35 Hz, 0.3 g, 20 min/day)	OVX osteoporotic fracture model	Macrophages	Restores early pro-inflammatory response and accelerates M2 transition	Improved callus formation and fracture healing	300
	Nanovibrational stimulation (~40 nm, 1 kHz)	3D human osteoblast/osteoclast co-culture	Osteoclast precursors	Akt-TGF-β1/BMP2 signaling mediates nanovibration response, reducing osteoclast activity	Enhanced osteogenesis and reduced osteoclastogenesis	301
	Distraction osteogenesis	Rat/rabbit/sheep models	Macrophages, T/B cells	Integrin-FAK-PI3K/AKT-MAPK-YAP/BMP2 pathways regulate M1-to-M2 transition	Controlled inflammation, neovascularization, and bone regeneration	303
Scaffold-based mechanical modulation	80 kPa polyacrylamide hydrogel	Mouse calvarial defect model	Macrophages	Inhibition of Piezo1 attenuates macrophage senescence and the pro-inflammatory phenotype, promotes M2 polarization through reduced IL-1β, IL-6, and TNF-α, and increased IL-10 and VEGF expression	Anti-inflammatory response, enhanced angiogenesis and osteogenesis, improved bone regeneration	315
	Low-stiffness Ti2448 scaffold	Rabbit; RAW264.7, HUVECs, MC3T3-E1	Macrophages	Active Piezo1/YAP axis modulates M2 polarization	Promotes angiogenesis & osteogenesis	316
	Nano-sheet array (Ti surface topography)	RAW264.7 cells	Macrophages	Activation of the integrin β2-FAK-PI3K-AKT-mTOR pathway promotes M2 polarization and upregulates TGF-β, BMP2, and VEGF	Suppressed inflammation, enhanced osteogenesis and angiogenesis	317
	Left-handed chiral nanohydroxyapatite (nHA) scaffolds	Tibial distraction osteogenesis model	Macrophages	L-chiral structures activate the integrin β1-FAK-TRPV4-Ca²⁺-STAT6 pathway to induce M2 polarization	Promoted vascularization and bone regeneration	318
	Aligned PCL nanofiber scaffold	Mouse Achilles tendon injury model	Macrophages	Scaffold alignment guides macrophage orientation, suppresses M1 polarization, and enhances M2 polarization with increased IL-10 and TGF-β secretion	Enhanced extracellular matrix synthesis and tenocyte activity, improved tendon healing	319
Mechanosensitive materials	Injectable piezoelectric hydrogel (PVA/CNF/BTO@PDA)	Rabbit rotator cuff injury model	Macrophages	Piezoelectric microelectrical cues enhance M2 polarization (elevated CD206, reduced iNOS) and recruit bone marrow stem cells for zonal differentiation	Enhanced fibrocartilage and bone formation, improved tendon-bone healing strength	320
	Janus asymmetric piezoelectric adhesive	Rat rotator cuff repair model	Macrophages	TRPV1-Ca²⁺-cAMP/PKA signaling promotes M2 polarization and stimulates VEGF and TGF-β secretion	Reduced local inflammation, enhanced angiogenesis and fibrocartilage regeneration	321
	Magnetized nanocomposite hydrogel	Rat calvarial defect model	Macrophages	Magnetic mechanical cues activate the podosome-Rho-ROCK signaling pathway, inducing time-dependent M1-to-M2 transition	Sequential immune modulation and improved bone formation	322
	Piezoelectric biomimetic periosteum (BaTiO₃/MWCNT/collagen)	Mouse cranial bone defect model	Macrophages	Ultrasound-activated Piezo1 triggers Ca²⁺ influx, suppresses NF-κB (TRAF6/TLR) signaling, and induces Arg1 and IL-10 expression	Enhanced osteogenic differentiation of bone marrow stem cells, improved bone healing	290
	BaTiO₃/Ti6Al4V piezoelectric scaffold	Sheep cervical bone defect model	Macrophages	Electrical stimulation inhibits MAPK/JNK signaling and enhances oxidative phosphorylation and ATP synthesis, leading to M2 polarization	Reduced inflammation and enhanced bone regeneration	323
	Perfusion bioreactor system with electrospun membranes	Rat calvarial defect model	Macrophages	Shear stress activates Piezo1-Ca²⁺-YAP signaling, promoting M2 polarization and secretion of miR-210-3p-enriched extracellular vesicles	Increased extracellular vesicle yield (~12.5-fold), enhanced bone regeneration and angiogenesis	324

ACLT, anterior cruciate ligament transection; BAIBA, β-aminoisobutyric acid; BMP, bone morphogenetic protein; BTO, barium titanate; CCL3, chemokine (C-C motif) ligand 3; DMM, destabilization of the medial meniscus; ECM, extracellular matrix; FAK, focal adhesion kinase; IFN-γ, interferon-γ; IL, interleukin; JAK, Janus kinase; LIPUS, low-intensity pulsed ultrasound; LXA4, lipoxin A4; MCP-1, monocyte chemoattractant protein-1; MHC, major histocompatibility complex; MIA, monosodium iodoacetate; MWCNT, multi-walled carbon nanotube; NF-κB, nuclear factor-κB; NLRP3, NOD-like receptor protein 3; NRF2, nuclear factor erythroid 2-related factor 2; OA, osteoarthritis; OVX, ovariectomized; PCL, polycaprolactone; PKA, protein kinase A; PKM2, pyruvate kinase M2; PVA, polyvinyl alcohol; Rho, Ras homolog family; ROCK, Rho-associated protein kinase; SAMP8, senescence-accelerated mouse prone 8; SQSTM1, sequestosome-1/p62; STAT6, signal transducer and activator of transcription 6; TGF-β, transforming growth factor-β; Tregs, regulatory T cells; TRPV1/4, transient receptor potential vanilloid 1/4; VEGF, vascular endothelial growth factor; YAP, yes-associated protein

## Abbreviations

ADAMTS: a disintegrin and metalloproteinase with thrombospondin motifs
AFCs: annulus fibrosus cells
AKT: protein kinase B
BMMs: bone marrow macrophage
BMP2: bone morphogenetic protein 2
BMSCs: bone marrow stromal cells
CaMKII: calmodulin-dependent kinase II
CCL: CC chemokine ligand
DAMPs: damage-associated molecular patterns
DMM: destabilization of the medial meniscus
DRG: dorsal root ganglia
ECM: extracellular matrix
EVs: extracellular vesicles
FAK: focal adhesion kinase
IDD: intervertebral disc degeneration
IGF-1: insulin-like growth factor 1
IL-17A: interleukin-17A
K2P: two-pore potassium channels
LCS: lacunocanalicular system
LINC: linker of nucleoskeleton and cytoskeleton
LIPUS: low-intensity pulsed ultrasound
L-BAIBA: L-β-aminoisobutyric acid
MAPK: mitogen-activated protein kinase
MMP-9: matrix metalloproteinase-9
MSCs: mesenchymal stem cells
NGF: nerve growth factor
NFAT: nuclear factor of activated T cells
NF-κB: nuclear factor kappa-light-chain-enhancer of activated B cells
NLRP3: NOD-like receptor family pyrin domain containing 3
OA: osteoarthritis
OPG: osteoprotegerin
OP: osteoporosis
Piezo1: piezo-type mechanosensitive ion channel component 1
PDGF-BB: platelet-derived growth factor-BB
PI3K: phosphatidylinositol 3-kinase
RANKL: receptor activator of nuclear factor κB ligand
RCN2: reticulocalbin-2
Rho-ROCK: Ras homolog-Rho-associated coiled-coil kinase
SASP: senescence-associated secretory phenotype
SIRT6: sirtuin 6
STAT: signal transducer and activator of transcription
TCR: T cell receptor
TGF-β1: transforming growth factor-β1
Th17: T helper 17 cells
Tregs: regulatory T cells
TRPV4: transient receptor potential vanilloid 4
VEGF-C: vascular endothelial growth factor-C
VEGFR3: vascular endothelial growth factor receptor 3
WBV: whole body vibration
Wnt3a: Wingless-type MMTV integration site family member 3A
YAP/TAZ: Yes-associated protein/transcriptional coactivator with PDZ-binding motif
NPCs: nucleus pulposus cells
